# A Post-Encapsulation Method for the Preparation of mRNA-LNPs via the Nucleic Acid-Bridged Fusion of mRNA-Free LNPs

**DOI:** 10.1021/acs.nanolett.4c06643

**Published:** 2025-04-12

**Authors:** Hiroki Tanaka, Yuka Sato, Tomoya Nakabayashi, Akari Tanaka, Kazuma Nishio, Chika Matsumoto, Atsuya Matsumaru, Takuma Yamakawa, Kota Ishizaki, Keisuke Ueda, Kenjirou Higashi, Kunikazu Moribe, Yuta Nakai, Kota Tange, Hidetaka Akita

**Affiliations:** † Laboratory of DDS Design and Drug Disposition, Graduate School of Pharmaceutical Sciences, Tohoku University, 6-3, Aoba, Aramaki, Aoba-ku, Sendai city, Miyagi 980-8578, Japan; ‡ Center for Advanced Modalities and DDS, Osaka University, Suita 565-0871 Osaka, Japan; § Laboratory of DDS Design and Drug Disposition, Graduate School of Pharmaceutical Sciences, Chiba University, 1-8-1, Inohana, Chuo-ku, Chiba city, Chiba 260-0856, Japan; ∥ Laboratory of Pharmaceutical Technology, Graduate School of Pharmaceutical Sciences, Chiba University, 1-8-1, Inohana, Chuo-ku, Chiba city, Chiba 260-0856, Japan; ⊥ Life Science Research Laboratory, NOF Corporation, 3-3 Chidori-cho, Kawasaki-ku, Kawasaki city, Kanagawa 210-0865, Japan

**Keywords:** Lipid nanoparticles, Gene therapy, mRNA vaccines, Drug delivery systems

## Abstract

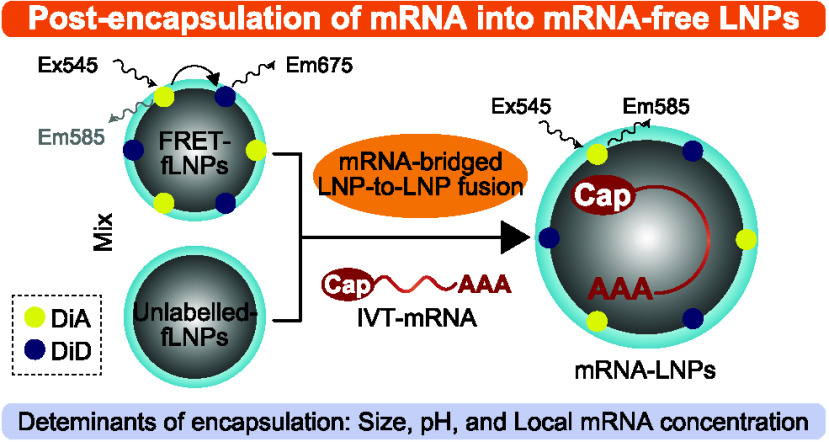

Lipid nanoparticles with encapsulated mRNA (mRNA-LNPs) have become key modalities for personalized medicines and RNA vaccines. Once the platform technology is established, the mRNA-LNPs could be applicable to a variety of protein-based therapeutic strategies. A post-encapsulation method, in which the mRNA solution is incubated with preformed mRNA-free LNPs to prepare the mRNA-LNPs, would accelerate the development of RNA-based therapeutics since even nonexperts could manufacture the mRNA-LNPs. In this study, we describe that the post-encapsulation of mRNA into mRNA-free LNPs is accompanied by “nucleic acid-bridged fusion” of them. The adsorption of mRNA onto mRNA-free LNPs via electrostatic interactions and the internalization of mRNA into the LNPs via particle-to-particle fusion are two steps that occur at different levels of pH. To complete post-encapsulation using only one-step mixing, the pH must be controlled within a limited region where both processes occur simultaneously. The size of the mRNA-free LNPs determines the effectiveness of mRNA loading.

Therapeutics based on nucleic acids are a fundamental technology for realizing rapid practical application of personalized medicines and vaccines. Since nucleic acids such as DNA and RNA are hydrophilic and anionic materials, they cannot penetrate the plasma membrane without help. Therefore, a drug delivery system (DDS) is required to deliver the drug to their site-of-action in cells. Lipid nanoparticles (LNPs) have been extensively studied for the delivery of RNA molecules and already used in the clinical situation.^1−5^ LNPs generally consist of ionizable lipids, helper phospholipids, sterols, and polyethylene glycol-conjugated lipids (PEG-Lipids).^6^ An ionizable lipid with tertiary amine(s) in its structure is a key material for the cytoplasmic delivery of RNAs,^7^ while other lipids confer stability. When the LNPs are taken up by cells, the tertiary amines in the ionizable lipids develop a cationic charge in the acidic environment of endosomes and promote the endosomal escape process of their cargo.^8^

When the LNPs are manufactured, lipids in ethanol and RNAs in an acidic buffer are mixed with a microfluidic mixer.^9−13^ This ethanol dilution method is the current gold standard for LNP production. The resultant formulations are subjected to a buffer replacement process for replacing the mixture of acidic buffer and ethanol with physiological buffers at a neutral pH. Filtration methods such as dialysis, ultrafiltration, or tangential flow filtration (TFF) are generally employed for this purpose.^14,15^

In early reports of the siRNA-loading LNPs (siRNA-LNPs), it was assumed that LNPs with inverted micelle-like structures housing siRNA were formed by the dilution process.^16^ It has been suggested that RNAs and lipids form complexes via electrostatic interactions on the order of one hundred milliseconds during this dilution.^17^ On the other hand, a recent report revealed that preformed LNPs and siRNA successfully formulated siRNA-LNPs via a microfluidic mixer.^18^ Researchers also recognized that the formation of LNPs is completed via mutual fusion of particles when the surface cationic charges of the LNPs were neutralized by pH increase.^19−21^ A two-step TFF, in which ethanol removal and the pH increase were separately conducted, produces more homogeneous and stable mRNA-LNPs.^22^ These examples indicate that an understanding of the LNP formation mechanisms is beneficial when attempting to establish an alternative method for producing LNPs.

As an alternative mRNA-LNP formulation method, we previously developed a ready-to-use type of freeze-dried LNPs (LNPs(RtoU/FD)) for the post-encapsulation of mRNA.^23^ Researchers can prepare mRNA-LNPs by adding a solution of their mRNA to freeze-dried mRNA-free LNPs with a short incubation at more than 75 °C. It has only been clarified that the mechanism for post-encapsulation involves changes in fluidity by heating. The requirement of heating, however, has raised concerns about the possibility of the deterioration of the mRNA.

In this study, we focused on a simpler principle of the mRNA-LNP formulation, which is termed liquid-type ready-to-use LNPs (LNPs(RtoU/Liq)) and allows users to formulate mRNA-LNPs simply by mixing aqueous solutions of mRNA with an aqueous suspension of mRNA-free LNPs. We found that the post-encapsulation process of mRNAs into preformed mRNA-free LNPs is accompanied by two sequential processes: “mRNA adsorption” and subsequent “nucleic acid-bridged LNP fusion”. More importantly, we found that these processes can be simultaneously run when the pH is adjusted to a limited range.

## The Efficiency of Post-Encapsulation Depends on pH and Lipid Composition

**Figure 1:**
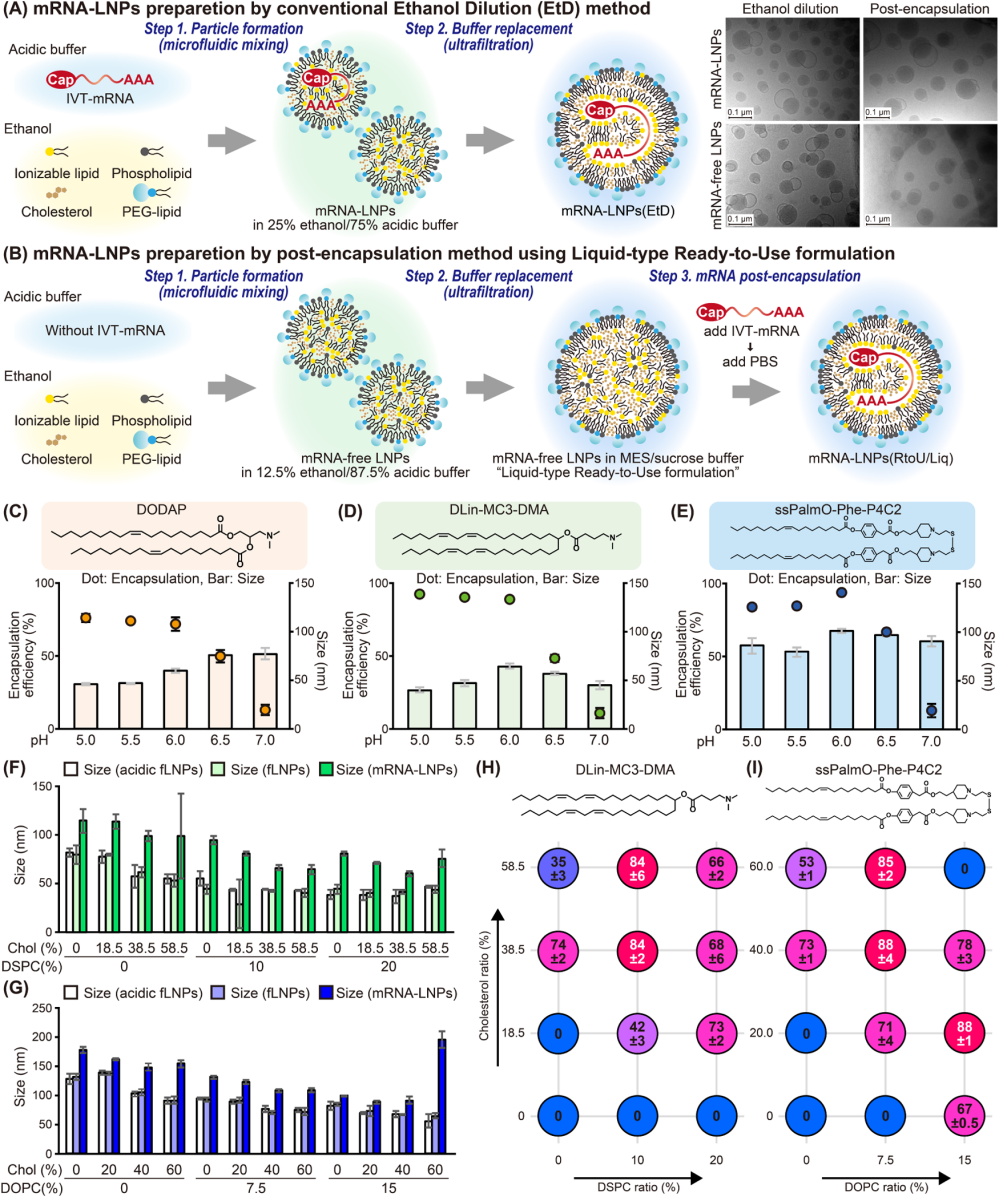
Proof of concept for the post-encapsulation method. (A, B) Schematic diagram illustrating the ethanol-dilution method (“EtD”, A) and the post-encapsulation method using a liquid-type, ready-to-use formulation (“RtoU/Liq”, B). (C–E) Postencapsulation of mRNA using DODAP, DLin-MC3-DMA, and ssPalmO-Phe-P4C2. (F, G) Effects of lipid compositions on the size of LNPs containing DLin-MC3-DMA (F) and ssPalmO-Phe-P4C2 (G). The particle sizes of mRNA-free LNPs before mRNA addition (acidic-fLNPs), neutralized mRNA-free LNPs without mRNA addition (fLNPs), and mRNA-LNPs formulated by post-encapsulation are shown. (H, I) Effects of lipid compositions on the encapsulation efficiency of LNPs containing DLin-MC3-DMA (H) and ssPalmO-Phe-P4C2 (I).

Schematic illustrations of the ethanol dilution (EtD) method used to produce mRNA-LNPs(EtD) and the post-encapsulation method for mRNA-LNPs(RtoU/Liq) appear in Figures 1A and 1B, respectively. In the post-encapsulation method, mRNA-free LNPs(RtoU/Liq) were first obtained via microfluidic mixing in the absence of mRNA. The buffer was subsequently replaced with a 2-(*N*-morpholino)ethanesulfonic acid (MES)/sucrose buffer. To prepare the mRNA-LNPs(RtoU/Liq), mRNAs in the same MES buffer were added to the mRNA-free LNPs(RtoU/Liq) under vortex mixing (Movie S1). One of the most important conclusions from this study is that the pH must be adjusted to approximately 6.0 for this post-encapsulation process. The mixture was incubated at 37 °C for 5 min to minimize deviations among the experiments, while it will be shown later that this incubation process can be omitted. Phosphate-buffered saline (PBS) was then added to neutralize the pH.

The post-encapsulation method was applied to three types of ionizable lipids: DODAP,^9^ DLin-MC3-DMA,^24^ and ssPalmO-Phe-P4C2.^25^ The lipid compositions of DODAP and DLin-MC3-DMA consisted of ionizable lipid/distearoyl-*sn*-glycero phosphatidyl choline (DSPC)/cholesterol/1-(monomethoxy polyethylene glycol 2000)2,3-dimyristoylglycerol (DMG-PEG2000) in a molar ratio of 50/10/38.5/1.5, which has been adopted for use in various ionizable lipids. The lipid composition for ssPalmO-Phe-P4C2 was ionizable lipid/dioleoyl-*sn*-glycero phosphatidyl choline (DOPC)/cholesterol/DMG-PEG2000 in a molar ratio of 52.5/7.5/40/1.5, which confers the ability of expression as well as low levels of inflammatory properties.^23,25,26^ The lipid/RNA ratio (L/R ratio, nmol/μg) was used to explain the relative amount of total lipids to mRNA. A commercially available luciferase-mRNA was encapsulated at L/R 100, which equates to a nitrogen/phosphate ratio (N/P ratio) of approximately 16 for the DODAP and DLin-MC3-DMA. The encapsulation efficiency of mRNA was measured using the Ribogreen assay and is expressed as the percentage of mRNA shielded from the external fluid, as determined by its inaccessibility to a hydrophilic fluorescent dye.

To examine the effect of pH on the mixing step (step.3 in Figure 1B) during post-encapsulation, the mRNA and mRNA-free LNPs were mixed with pH conditions that ranged from 5.0 to 7.0 (Figure 1C, 1D, and 1E). All three ionizable lipids showed more than 70% encapsulation efficiency at pH 6.0 or lower. The p*K*_a_ values of mRNA-free LNPs measured by TNS binding assay^27^ were 5.5, 6.1, and 6.4 for DODAP, DLin-MC3-DMA, and ssPalmO-Phe-P4C2, respectively (Figure S1). On the other hand, the zeta potential of these mRNA-free LNPs was increased by lowering the pH from pH 7.0 (0 mV) to pH 5.0 (+35 mV) in a similar manner (Figure S2). Although the efficiency of post-encapsulation was not affected by types of buffers tested, it tended to decrease when the ionic strength of the buffer increased (Figure S3). These results indicate that the adsorption of mRNA to the LNP surface via electrostatic interactions is an indispensable step for post-encapsulation, and the zeta potential of +10 mV could be a threshold.

The effect of lipid compositions on the post-encapsulation process was analyzed for DLin-MC3-DMA and ssPalmO-Phe-P4C2. The LNPs mixed with mRNA were larger than those of mRNA-free LNPs (Figures 1F, 1G, and S4). Considering that all mRNA-free LNPs have a slight cationic charge at pH 6.0 with an average of +15.2 mV (Tables S1 and S2), an increase in the particle size was induced by the electrostatic interaction of mRNA and LNPs. For efficient encapsulation, approximately 20–40% of cholesterol and 7.5–20% of phospholipids proved essential (Figure 1H and 1I). Since these molecules are the main components of the outermost layer of mRNA-LNPs,^28,29^ it is assumed that they are needed to ensure that mRNA remains inside the LNPs following loading.^6,19^

### Encapsulation of mRNA Coupled to an Increase in Particle Size

**Figure 2:**
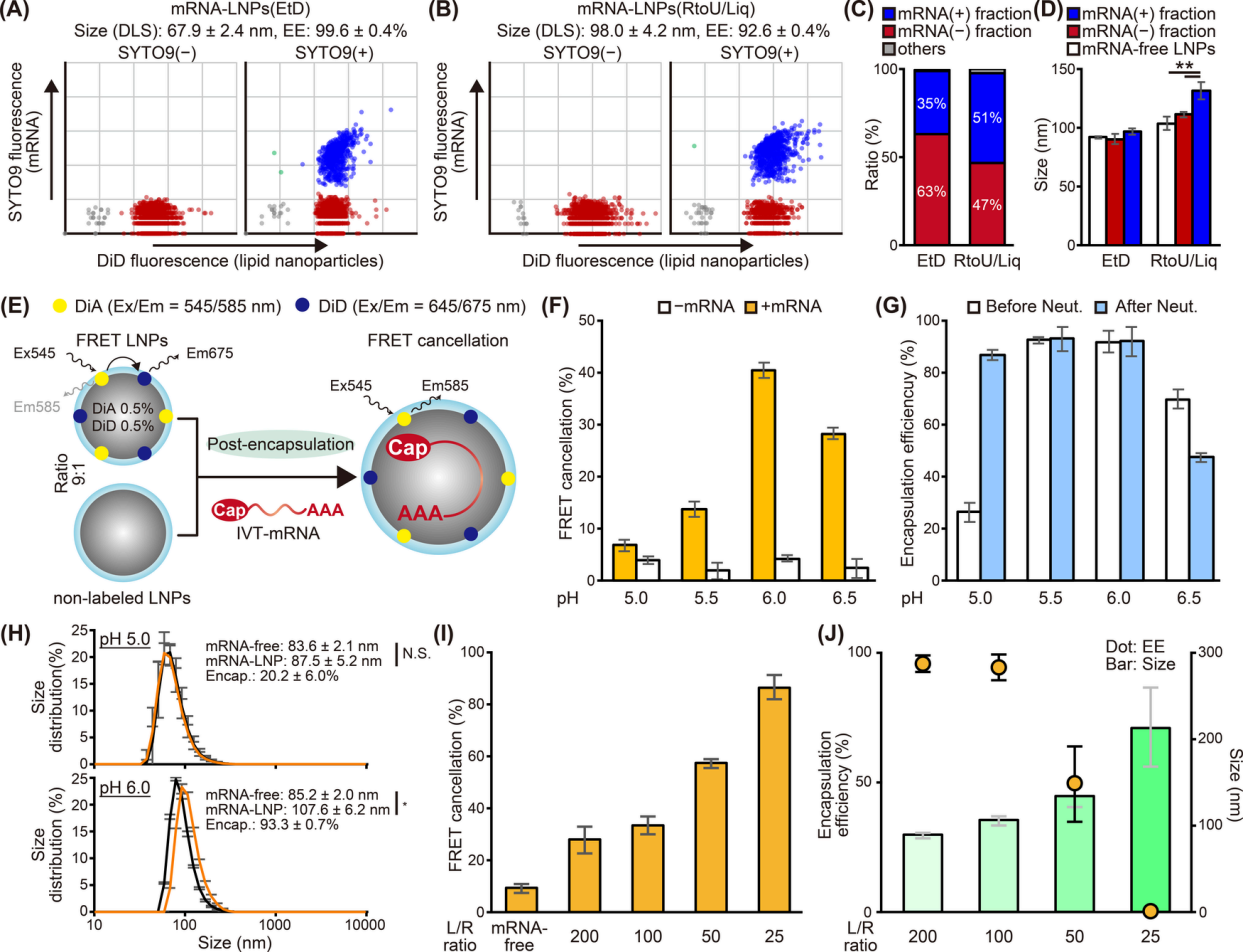
Nucleic acid-bridged LNP-to-LNP fusion induced by mRNA. (A–D) Single-particle analysis of mRNA-LNPs. mRNA-LNPs prepared using either the ethanol dilution method (A) or the post-encapsulation method (B) were analyzed using NanoFCM. Percentages of the events in each fraction are summarized (C). The size of the LNPs in each fraction was calculated using the beads calibration method (D). Statistical analysis was performed using one-way ANOVA followed by a Tukey’s multiple comparisons test, **p < 0.01. (E, F) FRET cancellation assay. FRET LNPs labeled with the pair of DiA and DiD were mixed with nonlabeled LNPs at a ratio of 1:9. The recovery of the DiA fluorescence after the addition of mRNA was measured (E). Nucleic acid-bridged fusion by mRNA at the indicated pH is summarized (F). (G) Effects of neutralization. The encapsulation efficiency of mRNA was measured before and after PBS addition. (H) Size change of LNPs by mRNA addition. The mRNA-free LNPs(RtoU/Liq) were mixed with mRNA at pH 5.0 or pH 6.0. The size of the particles was measured using dynamic light scattering. (I, J) Properties of particles at different L/R ratios. The mRNA-LNPs were prepared at different L/R ratios by varying the mRNA amount at pH 6.0. Values for FRET cancellation (I), size, and encapsulation efficiency (J) were obtained.

The mRNA-LNPs(EtD) and mRNA-LNPs(RtoU/Liq) composed of ssPalmO-Phe-P4C2 were labeled with DiD. The size and ratio of either the mRNA(−) fraction or the mRNA(+) fraction in the same mRNA-LNP formulation were investigated via single-particle analysis using NanoFCM (Figures 2A, 2B, and S5).^21^ The mRNA(+) fractions were 35 ± 1% and 51 ± 4% for mRNA-LNPs(EtD) and mRNA-LNPs(RtoU/Liq), respectively (Figure 2C). In the case of mRNA-LNPs(EtD), the sizes of the mRNA(+) fraction and mRNA(−) fraction in the mRNA-LNP formulation were comparable to those of the mRNA-free LNPs (Figure 2D). This observation supports the understanding that the final size of LNPs prepared via ethanol dilution is determined by the maturation of LNPs during buffer replacement.^19−22^ On the other hand, in the case of mRNA-LNPs(RtoU/Liq), the particle size of the mRNA(+) fraction was significantly larger than that found in either the mRNA(−) fraction or mRNA-free LNPs. This result suggests that the processes of mRNA encapsulation and increasing in particle size are coupled in the post-encapsulation method.

### Nucleic Acid-Bridged LNP Fusion Occurs during Post-Encapsulation

The successful post-encapsulation (>70% encapsulation efficiency) increased the particle size by 1.27- to 1.78-fold (Figures 1H, 1I, S4, and 2D), which corresponds to a 2.0- to 5.6-fold increase in the particle volume. Since this large increase in particle volume is difficult to explain by the contribution of encapsulated mRNA alone, we hypothesized that particle-to-particle fusion of LNPs is also involved in this size increase. To verify this hypothesis, the fusion of LNPs after the addition of mRNA was investigated via Förster resonance energy transfer (FRET) using the combination of a FRET pair: DiA and DiD.^20,25^ These dually labeled mRNA-free LNPs were mixed with unlabeled LNPs with a lipid molar ratio of 1:9. FRET cancellation was evaluated by measuring the recovery of DiA fluorescence following the addition of mRNA (Figure 2E). The addition of mRNA induced the fusion of LNPs, which we refer to as “nucleic-acid-bridged LNP-to-LNP fusion”, at pH 6.0 (Figure 2F). This fusion significantly enhanced the encapsulation of mRNA at this pH (Figure 2G). FRET cancellation was decreased at pH 6.5 probably due to a decrease in the electrostatic interactions between mRNA and the mRNA-free LNPs (Figure S2) and was consistent with the low encapsulation efficiency at this pH (Figures 1C, 1D, 1E, and 2G). Unexpectedly, the FRET cancellation also decreased at a pH lower than 6.0. This indicates that the fusion of LNPs could not occur at a pH of 5.0, though the mRNAs are probably adsorbed onto the positively charged mRNA-free LNPs. Consistent with this observation, the encapsulation of mRNA was <30% when measured at this pH. It is noteworthy that the encapsulation efficiency recovered when the pH was neutralized by adding PBS (Figure 2G). The size distribution of LNPs was shifted toward larger sizes by 1.26-fold at pH 6.0 with the addition of mRNA, while it remained unchanged at pH 5.0 with the addition of mRNA (Figure 2H). These observations suggest that even when mRNA interacts with LNPs at an acidic pH, the pH should be optimized to 5.5–6.0 to permit the fusion of LNPs to occur. At pH 5.0, encapsulation of mRNA via LNP-to-LNP fusion likely occurred during the subsequent neutralization step.

To investigate the stoichiometry of mRNA and LNPs, FRET cancelation was investigated by varying the L/R ratio at pH 6.0. As a result, the encapsulation efficiency was drastically decreased in an L/R below 50, whereas increasing the relative mRNA amount similarly enhanced the FRET cancellation and particle size (Figure 2I,J and Figure S6). To examine the state of the mRNA, electrophoresis was performed (Figure S7). In the case of L/R 100, which showed an encapsulation efficiency of 81% with the Ribogreen assay, only 11% of the total nucleic acids were detected as free mRNA. In the case of the L/R 25 sample, which has an encapsulation efficiency of 0% as determined using the Ribogreen assay, 62% of the total nucleic acids were detected as free bands. These results suggest that a large portion of the unencapsulated mRNA are present as free mRNA at a low L/R ratio. However, the residual 38% is released after particle dissolution with surfactant, suggesting that a part of mRNA was forming a complex with lipids. These results indicated that the mRNA interacting with LNPs at a low L/R was accessible even after the LNP-to-LNP fusion. Since mRNA-LNPs(EtD) could be prepared with these L/R ratios (Table S3), the decrease in the encapsulation efficiency could have originated from the mechanism of post-encapsulation.

To investigate the effects of mixing conditions, the volume ratio of the dispersion of mRNA-free LNPs and mRNA solution was varied while maintaining a constant final L/R ratio and concentration (Table S4). Two L/R ratios, 50 or 100, were used to examine the effects of their relative amounts. In this mixing process, the mRNA solution was added to the LNP dispersion in a tube. Vortex mixing and gentle pipetting were used as rapid and slow mixing methods, respectively. As a result, the encapsulation efficiency was decreased when a high concentration of mRNA was added to the LNP solution particularly in slow mixing. The size distribution of these particles suggests the presence of larger particles (Figure S8). Therefore, it is plausible that exposure of LNPs to a high concentration of mRNA resulted in the failure of post-encapsulation. The rapid mixing is a key factor for successful post-encapsulation in order to avoid the long-term exposure of LNPs to high concentrations of mRNA.

### Liquid-Type Ready-to-Use LNPs Can Be Used for Both *in Vivo* and *in Vitro* mRNA Delivery

**Figure 3:**
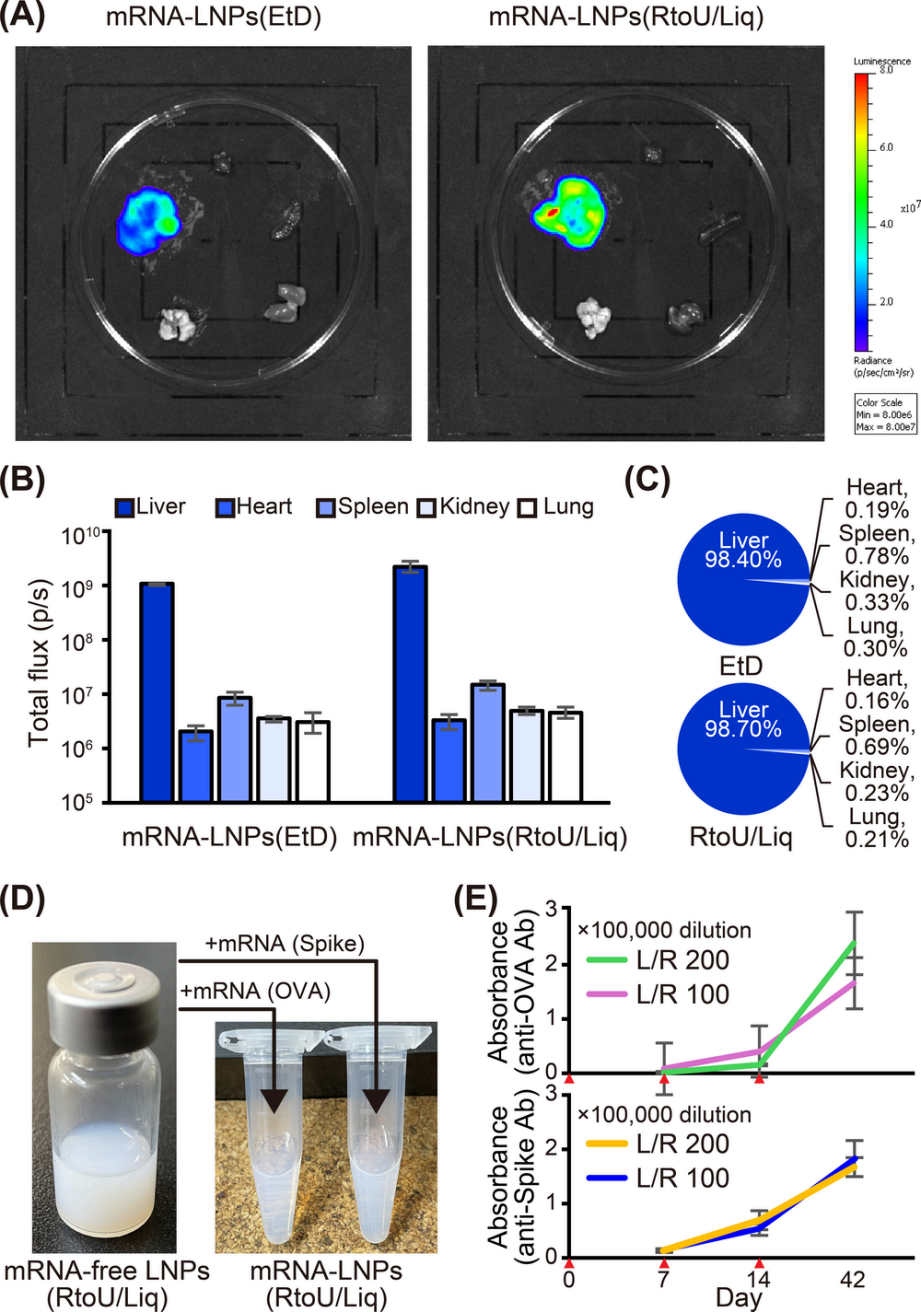
*Invivo* mRNA delivery using mRNA-LNPs(RtoU/Liq). (A–C) Expression of luciferase mRNA after intravenous injection. mRNA-LNPs(EtD) and mRNA-LNPs(RtoU/Liq) were injected to BALB/c mice at 0.1 mg/kg via the tail vein. At 6 h after the injection, the luminescence of luciferase was evaluated using the *invivo* imaging system, IVIS (A). The distribution of the expression from excised tissues was obtained (B) and summarized (C). (D, E) mRNA vaccine application of mRNA-LNPs(RtoU/Liq). mRNA encoding chicken ovalbumin (OVA) or spike protein of SARS-CoV-2 (SARS-CoV2-Spike) was formulated with the mRNA-free LNPs(RtoU/Liq) (E). The particles were intramuscularly administered to C57BL/6J mice at 1.5 μg/head three times in two-week intervals. The timing of administration is indicated by red arrowheads. At 2 weeks after each injection, blood was collected. Total IgG titer against these antigens was evaluated via ELISA.

The mRNA-LNPs(EtD) and mRNA-LNPs(RtoU/Liq) containing luciferase mRNA were intravenously administered to BALB/c mice. mRNA-LNPs(RtoU/Liq) was prepared by post-encapsulation at pH 6.0 and then neutralized with PBS. Six hours following administration, luciferase activity was visualized using the *in vivo* imaging system, IVIS (Figure 3A). As a result, comparable liver-selective gene expressions (>98%) were detected for both mRNA-LNPs (Figures 3B and 3C). Both *in vivo* and *in vitro* gene expressions were not affected by the incubation temperature (Figures S9 and S10).

An mRNA vaccine is one application of mRNA-LNPs. As model antigens for the mRNA vaccines, mRNA encoded either with ovalbumin (OVA) or with a spike protein of severe acute respiratory syndrome coronavirus 2 (SARS-CoV-2-Spike) was formulated as mRNA-LNPs(RtoU/Liq) at pH 6.0 and then neutralized with PBS (Figure 3D). The particles were intramuscularly administered three times in two-week intervals. As a result, robust antibody production was confirmed by the repeated injections of mRNA-LNPs(RtoU/Liq) induced in these mice (Figure 3E).

**Figure 4:**
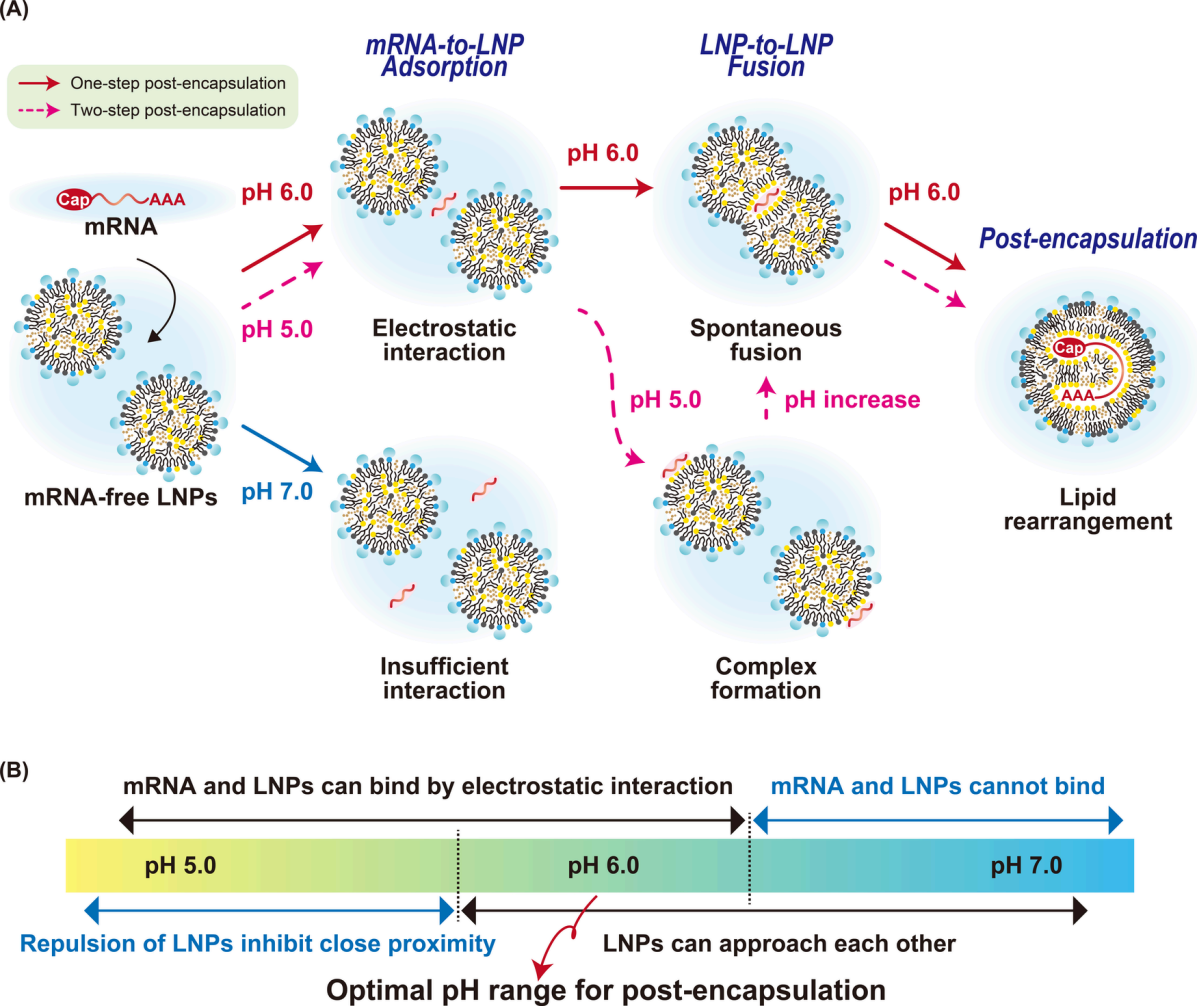
Schematic illustration of the post-encapsulation process. (A) The scheme of post-encapsulation. The post-encapsulation method was performed by adding a solution of mRNA to a dispersion of mRNA-free LNPs. When the solution had a neutral pH, the mRNA and LNPs could not interact. When the solution had a weakly acidic pH, the mRNA and LNPs interacted with each other. At pH 5.0, the complex of mRNA and LNPs seemed stable. On the other hand, at pH 6.0, the mRNA caused a bridging of the mRNA-free LNPs and subsequent LNP-to-LNP fusion. (B) Optimal pH range for post-encapsulation. Post-encapsulation requires both “interactions between mRNA and LNP” and “interactions between LNPs”. Since these phenomena occurred at different, but partially overlapped, values of pH, the post-encapsulation could be induced within a limited pH range of approximately 6.0 via simple one-step mixing. It should be noted that the intermediate pH was omitted for simplicity.

The schematic diagram in Figure 4A illustrates the post-encapsulation process. The adsorption of mRNA and the subsequent fusion of LNPs have different pH requirements. LNPs adsorb mRNA at pH 6.0 or below, where the zeta potential of LNPs is >10 mV (Figure S2). On the other hand, the mRNA-bridged LNP-to-LNP fusion occurs at a limited pH range of approximately pH 6.0. It has been known that the empirical threshold of zeta potential for colloidal particles to stably exist is ±30 mV.^20,30,31^ Therefore, electric repulsion could prevent mutual collision and unification of the particles at pH 5.0 since the particles showed a zeta potential of more than +30 mV even after the addition of mRNA (Figure S2). It is worth noting that these mRNA-free LNPs remained stabilized by the steric hindrance of DMG-PEG2000. In fact, the modification of mRNA-free LNPs with a high amount of DMG-PEG2000 (more than 3.75%) inhibited the post-encapsulation process (Figure S11). These observations suggest that the post-encapsulation process via LNP-to-LNP fusion occurs only when the mRNA-mediated bridging of LNPs overcomes the electrostatic repulsion and/or steric hindrance between the LNPs. In other words, post-encapsulation required both “interactions between mRNA and LNPs” and “interactions between LNPs themselves” (Figure 4B). For the purpose of accelerating mRNA drug discovery, one-step post-encapsulation at a pH of approximately 6.0 is optimal, since the steps of the formulation process should be minimized to allow use by nonexpert scientists.

The intraparticle microstructure of LNPs varies depending on the pH, temperature, and the presence of nucleic acids.^28,32^ In the absence of nucleic acids, the internal hydrophobic phase of LNPs showed an inverse hexagonal phase (H_II_) and an inverse micelle phase (L_II_) below pH 5.0 and above pH 7.0, respectively.^28^ In the intermediate region around pH 6.0, an inverse micellar *Fd*3*m* cubic phase was observed.^33^ On the other hand, in the presence of long nucleic acids such as mRNA or poly A, these lipids are supposed to coexist as a complexed inverse hexagonal phase (H_II_c), a complexed inverse micelle phase (L_II_c), and/or an inverse worm-like micelle phase.^28,33^ Therefore, a dynamic rearrangement of LNP structure such as phase transition is expected to be necessary to complete the post-encapsulation via fusion.^34,35^ A high level of mobility of the lipid molecules might be required to accomplish these processes. In the previous LNPs(RtoU/FD), low temperature and/or water removal during the freeze-drying process induced a decrease in the fluidity of the hydrophobic chains in LNPs.^23^ In that case, heating of the formulation was required for the completion of post-encapsulation. By contrast, the fluidity of the hydrophobic chains in LNPs(RtoU/Liq) was comparable to that in LNPs(EtD) (Figure S12). This observation explains the high level of encapsulation efficiency for the mRNA-LNPs(RtoU/Liq) even at room temperature.

In the range of conditions examined in this study, it is likely that “the nucleic acid-bridged LNP-to-LNP fusion” could be a necessary, but not a sufficient, condition for “successful post-encapsulation”. Analysis of the effects of lipid composition suggests that stabilization of the outer layers is required for keeping mRNA inside (Figure 1H and 1I). In addition, increases in the amount of mRNA (L/R 50 or L/R 25) resulted in the further fusion of LNPs, in parallel with an increase in size (Figure 2I), while the encapsulation efficiency was drastically decreased (Figure 2J). In these conditions with excessive mRNA amounts, large portions of mRNA molecules were present as free mRNA without interaction with LNPs. However, our data indicate that a portion of mRNA interacts with the surface of LNPs at low L/R ratios, where small molecules (Ribogreen reagent) can access. It should be noted that even when the loading amount of LNPs was sufficient from the viewpoint of the L/R ratio, the encapsulation efficiency was also decreased when a high concentration of the mRNA solution was mixed with mRNA-free LNPs and/or when the mRNA solution and mRNA-free LNPs were mixed slowly (Table S4 and Figure S8). Under these conditions, a portion of the LNPs interacts with large amounts of mRNA molecules during the mixing. Reports have noted that when cationic lipids interact with nucleic acids, the lipid molecules are segregated as a complex with the nucleic acids.^36^ Thus, it is possible that the extensive surface coverage by a high concentration of mRNA on mRNA-free LNPs could have resulted in a high level of lipid segregation, which would inhibit the phase transition of LNPs to a phase that suits mRNA encapsulation (i.e., H_II_c phase, L_II_c phase, and/or an inverse worm-like micelle phase) (Figure S13).

Given the hypothesis that low surface coverage is important for the post-encapsulation process, one strategy to improve the encapsulation efficiency at low L/R ratio could be reducing the size of mRNA-free LNPs to increase the specific surface area. To verify this hypothesis, 14 different sizes of mRNA-free LNPs with the same composition were prepared via membrane emulsification and microfluidic mixing. As a result, the encapsulation efficiency showed a strong negative correlation (*R* = −0.87) to the size of the particles before mRNA addition (Table S5 and Figure S14). The same tendency was observed when the mRNA-free LNPs were prepared via bulk mixing. Therefore, the smaller size of mRNA-free LNPs is important to the post-encapsulation process.

In conclusion, we reported the development of LNPs(RtoU/Liq) that could be used to prepare mRNA-LNPs simply by mixing them with mRNA on demand. Since heating is not required, the proposed method would have little effect on the mRNA quality. The pH of the mRNA-free LNP suspensions and the mRNA solution should be adjusted to pH 6.0, where the nucleic acid-bridged LNP-to-LNP fusion by mRNA occurs spontaneously. The efficiency of post-encapsulation is affected by the local concentration of mRNA and by the size of the mRNA-free LNPs. A more detailed analysis of the mode of the structural rearrangement and the dependency on the specific surface area will be an important challenge in the future.

## Materials and Methods

Experiment protocols with lists of reagents are summarized in the Supporting Information.

### Animal Experiments

C57BL/6J mice (C57BL/6JJmsSlc, female, 6–8 weeks) and BALB/c mice (BALB/cCrSlc, female, 6–8 weeks) were purchased from Japan SLC, Inc. (Shizuoka, Japan). The animals were maintained under a 12 h light/12 h dark cycle. The experimental protocols were reviewed and approved by the Tohoku University Animal Care Committee or the Chiba University Animal Care Committee in accordance with the “Guide for Care and Use of Laboratory Animals”.

### Statistics

Statistical analyses were performed using GraphPad Prism ver. 10.2.1 (395). The methods used for each analysis are described in the captions.

## Supporting Information Available

S1. List of materials used in the study, S2. Experiment protocols, S3. Sequence of pDNA template for vaccination, S4. Supplementary Figures (S1–S14), Tables (S1–S5) and Caption of Movie S1, S5. References for Supporting Information (PDF)
Movie S1: A demonstration of how mRNA-LNPs are prepared using the mRNA-free LNPs(RtoU/Liq) (MP4)
