# Supplementary material for: A Post-Encapsulation Method for the Preparation of mRNA-LNPs via the Nucleic Acid-Bridged Fusion of mRNA-Free LNPs
Source: Nano Lett. 2025 Apr 12;25(16):6445–53. doi: 10.1021/acs.nanolett.4c06643 (PMC12023019; doi:10.1021/acs.nanolett.4c06643)
Supplement: Supplementary file 1 — nl4c06643_si_001.pdf [file nl4c06643_si_001.pdf]

Supporting Information

Nano Letters

Title:

A post-encapsulation method for the preparation of mRNA-LNPs via the nucleic acid-bridged fusion of mRNA-free LNPs

Author:

Hiroki Tanaka,[1,2]* Yuka Sato,[3] Tomoya Nakabayashi,[1] Akari Tanaka,[1] Kazuma Nishio,[1] Chika Matsumoto,[1] Atsuya Matsumaru,[1] Takuma Yamakawa,[3] Kota Ishizaki,[3] Keisuke Ueda,[4] Kenjirou Higashi,[4] Kunikazu Moribe,[4] Yuta Nakai,[5] Kota Tange,[5] and Hidetaka Akita[1,2]*

Affiliations:

[1] Laboratory of DDS Design and Drug Disposition, Graduate School of Pharmaceutical Sciences, Tohoku University, 6-3, Aoba, Aramaki, Aoba-ku, Sendai city, Miyagi, 980-8578, Japan

[2] Center for Advanced Modalities and DDS, Osaka University, Suita 565-0871, Osaka, Japan

[3] Laboratory of DDS Design and Drug Disposition, Graduate School of Pharmaceutical Sciences, Chiba University, 1-8-1, Inohana, Chuo-ku, Chiba city, Chiba, 260-0856, Japan

[4] Laboratory of Pharmaceutical Technology, Graduate School of Pharmaceutical Sciences, Chiba University, 1-8-1, Inohana, Chuo-ku, Chiba city, Chiba, 260-0856, Japan

[5] Life Science Research Laboratory, NOF CORPORATION, 3-3 Chidori-cho, Kawasaki-ku, Kawasaki city, Kanagawa 210-0865, Japan

***Corresponding author:**

email address: hiroki.tanaka.e1@tohoku.ac.jp (H. Tanaka)

Tel.: +81-22-795-6832

email address: hidetaka.akita.e4@tohoku.ac.jp (H. Akita)

Tel.: +81-22-795-6831

Contents

S1. List of materials used in the study

S2. Experiment protocols

S3. Sequence of pDNA template for vaccination

S4. Supplementary Figures and Tables

S4-1. Figure S1. An apparent pKa of mRNA-free LNPs.

S4-2. Figure S2. Zeta potential of LNPs at each pH before and after mRNA addition.

S4-3. Figure S3. Effects of buffer components.

S4-4. Figure S4. Correlation between size increase and encapsulation efficiency.

S4-5. Figure S5. Additional dot plots of mRNA-LNPs(EtD) and mRNA-LNPs(RtoU/Liq).

S4-6. Figure S6. Cryo-TEM images.

S4-7. Figure S7. Electrophoresis of mRNA formulated at different L/R ratio.

S4-8. Figure S8. Size distribution of particles with different mixing procedures.

S4-9. Figure S9. Effect of 37 °C incubation on the *in vivo* gene expression.

S4-10. Figure S10. Effect of incubation temperature on *in vitro* gene expression.

S4-11. Figure S11. Effects of DMG-PEG2000 amount on post-encapsulation.

S4-12. Figure S12. Steady-state anisotropy measurement.

S4-13. Figure S13. Hypothesis for the unsuccessful post-encapsulation.

S4-14. Figure S14. Size dependency of post-encapsulation.

S4-14. Table S1. Particle properties of DLin-MC3-DMA LNPs.

S4-15. Table S2. Particle properties of ssPalmO-Phe-P4C2 LNPs.

S4-16. Table S3. Particle properties of LNPs with different L/R ratios.

S4-17. Table S4. Particle properties of LNPs with different mixing conditions

S4-18. Table S5. Particle properties of LNPs prepared by various conditions

S4-19. Caption of Movie S1

S5. References for Supporting Information

Other supporting materials:

Movie S1

S1. List of materials used in the study

Reagent	Size	Manufacturer	Product number
COATSOME® SS-OP (ssPalmO-Phe-P4C2)	1 g	NOF CORPORATION	
COATSOME® CL-8181DA (DODAP)	1g	NOF CORPORATION	CL-8181DA
DLin-MC3-DMA		Cayman chemical	34264
Ethanol 99.5%	500 mL	Nacalai tesque	14712-05
SUNBRIGHT® GM-020(DMG-PEG)	1 g	NOF CORPORATION	GM-020
COATSOME® MC-8181 (DOPC)	1 g	NOF CORPORATION	MC-8181
COATSOME® MC-8080 (DSPC)	1 g	NOF CORPORATION	MC-8080
Cholesterol Sigma Grade, ≥99%	5 g	SIGMA Aldrich	C8667-5G
MES	100 g	Nacalai tesque	02442-44
DL-Malic Acid	500 g	Nacalai tesque	21029-55
Phthalic Acid	25 g	Nacalai tesque	27814-62
Maleic Acid	25 g	Nacalai tesque	11736-82
Succinic Acid	25 g	Nacalai tesque	32406-52
DL-Tartaric Acid	25 g	Nacalai tesque	32703-52
Citric Acid, Anhydrous	500 g	Nacalai tesque	09109-85
Acetic Acid	500 mL	Wako	017-00256
Sodium Chloride	500 g	Nacalai tesque	31320-05
HEPES	500 g	DOJINDO LABORATORIES	342-01375
Sucrose	500 g	Nacalai tesque	30404-45
UltraPure™ DNase/RNase-Free Distilled Water	500 mL	Invitrogen™	10977023
D-PBS(-)	500 mL	Nacalai tesque	14249-24
Quant-iT™ RiboGreen® RNA reagent	1 mL	Invitrogen™	R11491
D-Luciferin Potassium Salt	1 g	FUJIFILM Wako Pure Chemical	126-05116

		Corporation	
EDTA・2Na	500 g	DOJINDO	345-01865
TritonX-100 TM	500 mL	Nacalai tesque	12969-25
DiD	10 mg	Thermo Scientific	D7757
DiA	25 mg	Thermo Scientific	D3883
SYTO9	100 µL	Thermo Scientific	S34854
Recombinant Spike proten	50 µg	Adipogen Life Sciences	AG-40B-0205-C050
Albumin from chicken white egg	250 mg	Sigma Aldrich	A2512-250MG
Polyoxyethylene Sorbitan Monolaurate (Tween 20)	500 g	Nacalai tesque	35624-15
Fetal Bovine Serum (FBS)	500 mL	Gibco	10270
Goat anti-Mouse IgG-Fc Fragment Antibody HRP Conjugated	1 mL	Bethyl Laboratories	A90-131P
TMB Solution	100 mL	Merck Millipore	CL07-100ML
1mol/l Sulfuric Acid (H_2SO_4)	500 mL	Nacalai tesque	95626-06
Sodium Hydrogen Carbonate ($NaHCO_3$)	500 g	Nacalai tesque	31212-25
Sodium Carbonate (Na_2CO_3)	500 g	Nacalai tesque	31310-35
Isoflurane Inhalation Anesthetic Solution	250 mL	Viatris	1119701G1092
AmiconUltra-4-100K Centrifugal Units	4 mL volume	Merck	UFC810096
AmiconUltra-4-100K Centrifugal Units	15 mL volume	Merck	UFC910096
6-(p-Toluidino)-2-naphthalenesulfonic acid sodium salt (TNS)	250 mg	Sigma-Aldrich	T9792-250MG
1,6-diphenyl-1,3,5-hexatriene (DPH)	1 g	Sigma-Aldrich	D208000-1G
CleanCap Fluc mRNA	1 mg	TriLink BioTechnologies	L-7202-1000
MEGAscript™ T7 Transcription Kit	1 kit	Thermo Fisher Scientific	AM1334

ScriptCap Cap 1 Capping System	1 kit	cellscript	C-SCCS1710
LiCl Precipitation Solution (7.5 M)	100 mL	Thermo Fisher Scientific	AM9480
poly(A) Tailing Kit	1 kit	Thermo Fisher Scientific	AM1350
AscI	500 units	New England BioLabs	R0558S
Cellulose	100 g	Sigma-Aldrich	C6288-100G
Poly-(α,β)-DL-aspartic acid sodium salt	100 mg	Sigma-Aldrich	P3418-100MG
Agarose S	500 G	NIPPON GENE	318-01195
Sodium lauryl sulfate (Sodium dodecyl sulfate: SDS)	500 G	Nacalai Tesque	08933-05
Tris-Borate-EDTA Buffer(10x)	1 L	Nacalai Tesque	35440-31
SYBR® Green II Nucleic Acid Gel Stain	1 mL	Takara	5770A

S2. Detailed experiment protocols

mRNA synthesis

mRNA encoding luciferase was purchased from TriLink BioTechnologies. mRNA encoding OVA and SARS-CoV-2-Spike was synthesized in-house. The sequence of templates is described in subsequent sections. A reaction mixture was prepared by mixing 5 μg pDNA-Luc solution, 5 μL Cut Smart buffer, and 1 μL Restriction enzyme (AscI), and Nuclease-free water was added to reach a volume of 50 μL. The mixture was incubated at 37 °C for 16 hours. 50 μL Nuclease-free water and 100 μL TE saturated phenol:$CHCl_3$ = 1:1 were added, then the mixture was stirred using a vortex mixer for 1 minute. The mixture was centrifuged (4 °C, 15,000 rpm, 10 minutes), then the aqueous layer (upper layer) was collected. Nuclease-free water was added to reach a volume of 100 μL, then 250 μL of 99.5% EtOH, 5 μL of 5 M NaCl, 0.5 μL of 20 mg/mL glycogen were added. The mixture was incubated in a −20 °C freezer for 15 minutes. After centrifugation (4 °C, 15,000 rpm, 15 minutes), the supernatant was removed. The pellet was washed by adding 200 μL of 70% EtOH. After centrifugation (4 °C, 15,000 rpm, 5 minutes), the supernatant was removed, then the pellet was dried for 3 minutes. The pellet was dissolved using 20 μL of Nuclease-free water, and the concentration of linearized DNA was measured. mRNA was transcribed according to the MEGAscript™ T7 Transcription Kit (AM1334) protocol. The working mixture was prepared by mixing 2 μL of 75 mM ATP solution, 2 μL of 75 mM CTP solution, 2 μL of 75 mM GTP solution, 1.5 μL of 100 mM m1ΨTP solution, 2 μL of 10x Reaction buffer, and 1μg of linearized DNA. Nuclease-free water was then added to reach a volume of 18 μL. After adding 2 μL of T7 Enzyme Mix, the mixture was incubated at 37 °C for 1 hour. After 1 μL of TURBO DNase was added, the mixture was incubated at 37 °C for 15 minutes. Then, 30 μL of Nuclease-free water and 30 μL of LiCl Precipitation Solution were added and incubated at −20 °C for 30 minutes. After centrifugation (4 °C, 20400 ×g, 15 minutes), the supernatant was removed. The pellet was washed by adding 200 μL of 70% EtOH. After centrifugation (4 °C, 20,400 × g, 5 minutes), the supernatant was removed, then the pellet was dried for 30 seconds. The pellet was dissolved using 20 μL of Nuclease-free water, and 480 μL of chromatography buffer was added (10 mM HEPES, 0.1 mM 2NA(EDTA), 125 mM NaCl, 16% EtOH, pH 7.2).

The residual dsRNA was removed as described in the literature [1]. A suspension of 0.2 g/mL cellulose was allowed to shake at 1,200 rpm for 10 minutes in a shaking incubator. A column was filled with 350 μL of the cellulose suspension. The column was centrifuged (25°C, 14,000 ×g, 60 s), and 500 μL of the chromatography buffer was added into the column. The column was shaken at 1,200 rpm at 25 °C for 5 minutes. The column was centrifuged (14,000 ×g, 60 s), and 500 μL of the IVT-mRNA solution described above was added. The column was shaken at 1,200 rpm at 37 °C

for 30 minutes. The column was centrifuged and the eluted solution was collected into a 1.5 mL tube, then 50 µL of 3 M NaOAc and 500 µL of isopropanol were added. The mixture was incubated at -20 °C for 20 minutes. After centrifugation (4 °C, 15,000 × g, 10 minutes), the supernatant was removed. The pellet was washed by adding 200 µL of 70% EtOH. After centrifugation (4 °C, 20,400 × g, 5 minutes), the supernatant was removed, and the pellet was dissolved using 70 µL of Nuclease-free water.

The 5’ Cap was added according to the protocol of ScriptCap Cap 1 Capping System (C-SCCS1710). The solution of the IVT-mRNA was diluted to 100 µg/140 µL using Nuclease-free water. The solution was incubated at 65 °C for 5 minutes, then placed on ice while preparing the capping reagents mixture. The working mixture was prepared by mixing 20 µL of 10x ScriptCap Capping Buffer, 20 µL of 10 mM GTP, 5 µL of 20 mM SAM, 5 µL of ScriptGuard RNase Inhibitor, and 8 µL of ScriptCap 2’-O-Methyltransferase (100 U/µL). Then 8 µL of ScriptCap Capping Enzyme and 140 µL of the IVT-mRNA solution were added to the mixture. The mixture was incubated at 37 °C for 30 minutes, then 100 µL of LiCl Precipitation Solution was added. The mixture was incubated at −20 °C for 30 minutes. After centrifugation (4 °C, 20,400 × g, 15 minutes), the supernatant was removed. The pellet was washed by adding 200 µL of 70% EtOH. After centrifugation (4 °C, 20,400 ×g, 5 minutes), the supernatant was removed, then the pellet was dried for 30 seconds. The pellet was dissolved using 110 µL of Nuclease-free water.

The 3’ Poly(A) tail was added according to the protocol of poly(A) Tailing Kit (AM1350). The working mixture was prepared by mixing 110 µL of mRNA solution, 40 µL of 5x E-PAP Buffer, 20 µL of 25 mM $MnCl_2$, 20 µL of ATP Solution, and 8 µL of E-PAP Enzyme. The mixture was then incubated at 37 °C for 45 minutes. After the incubation, 100 µL of the LiCl Precipitation Solution was added and the mixture was then incubated at −20 °C for 30 minutes. After centrifugation (4 °C, 20,400 ×g, 15 minutes), the supernatant was removed. The pellet was washed by adding 200 µL of 70% EtOH. After centrifugation (4 °C, 20,400 × g, 5 minutes), the supernatant was removed, and the pellet was dried for 30 seconds. The pellet was dissolved using 50 µL of Nuclease-free water, and stored at −80 °C.

Preparation of LNPs(RtoU/Liq)

A stock solution of 10 mM of ionizable lipids (DODAP, DLin-MC3-DMA, or ssPalmO-Phe-P4C2), 10 mM of phospholipids (DSPC or DOPC), 10 mM of cholesterol, and 2 mM of 1-(Monomethoxy polyethyleneglycol2000)2,3-dimyristoylglycerol (DMG-PEG2000) was prepared in ethanol. Before the experiments, the lipid stocks were warmed to 32 °C for 10 minutes to eliminate the precipitation in the solution. These lipid stocks were mixed to prepare a lipid mixture as described in the main

text. The concentration of the lipid mixture in ethanol was 8 mM. The lipid mixture was mixed with a malic acid/NaOH buffer (20 mM, pH 3.0, without additional salts) without IVT-mRNA using a microfluidic mixer NanoAssemblr™. The total flow rate and FRR (water/ethanol) were 1 mL/min and 7/1, respectively. The resultant suspension of mRNA-free LNPs in the mixture of alcohol and malic acid buffer was diluted at least 4-fold by using MES/NaOH buffer (20 mM, indicated pH). The suspension was then transferred to Amicon Ultra-4-100K centrifugal units or to Amicon Ultra-15-100K centrifugal units according to the preparation scale. The suspension of mRNA-free LNPs was centrifuged for concentration (1,000 × g, room temperature). The resultant mRNA-free LNPs that remained in the upper cassette were re-diluted with the MES/NaOH buffer at least 10-fold and centrifuged again (1,000 × g, room temperature). This buffer replacement was repeated twice. The resultant suspension of mRNA-free LNPs was collected and diluted to 40 mM total lipids. Then, an equal volume of 320 mg/mL sucrose solution was added to prepare the mRNA-free LNPs(RtoU/Liq). For the encapsulation of mRNA, the mRNA-free LNPs(RtoU/Liq) were mixed with mRNA solution under vortex mixing. After incubation at 37 °C for 5 minutes, an equal volume of PBS(−) was added to the mixture. The size, polydispersity index (PdI), and zeta-potential of the LNPs were measured using a Zetasizer nano ZS and a Zetasizer Pro (Malvern Panalytical, Malvern, Worcestershire, UK).

Preparation of LNPs via ethanol dilution

A stock solution of 10 mM ssPalmO-Phe-P4C2, 10 mM DOPC, 10 mM cholesterol, and 2 mM DMG-PEG2000 was prepared in ethanol. Before the experiments, the lipid stocks were warmed to 32 °C for 10 minutes to eliminate the precipitation in the solution. These lipid stocks were mixed to prepare a lipid mixture shown in the main text. The concentration of the total lipid in ethanol was 4 mM. The lipid mixture was mixed with a malic acid/NaOH buffer (20 mM, pH 3.0, with 30 mM NaCl) with or without IVT-mRNA (for mRNA-free LNPs(EtD) and mRNA-LNPs(EtD), respectively) using the microfluidic mixer NanoAssemblr™. The Total flow rate and FRR (water/ethanol) were 4 mL/min and FRR = 3/1, respectively. The resultant suspension of LNPs in the mixture of alcohol and malic acid buffer was diluted by MES/NaOH buffer (20 mM, pH 6.5) at least 4-fold and transferred to Amicon Ultra-4-100K centrifugal units or Amicon Ultra-15-100K centrifugal units according to the preparation scale. The suspension of LNPs was centrifuged for concentration (1,000 × g, room temperature). The resultant LNPs that remained in the upper cassette were re-diluted with PBS(−) at least 10-fold and centrifuged again (1,000 × g, room temperature). This buffer replacement was repeated twice. The resultant suspension of LNPs(MF) was collected and diluted to adequate concentration.

Particle preparation by bulk mixing method and membrane emulsification method

The lipid composition of the mRNA-free LNPs was fixed to ssPalmO-Phe-P4C2/DOPC/cholesterol/DMG-PEG2000 = 52.5/7.5/40/1.5.

Bulk mixing method: Bulk mixing was performed using a vortex mixer. A stock solution of 10 mM lipids ssPalmO-Phe-P4C2, 10 mM DOPC, 10 mM cholesterol, and 2 mM 1-(Monomethoxy polyethyleneglycol2000)2,3-dimyristoylglycerol (DMG-PEG2000) was prepared in ethanol. Before the experiments, the lipid stocks were warmed to 32 °C for 10 minutes to eliminate the precipitation in the solution. These lipid stocks were mixed to prepare a lipid mixture. The lipid mixture was mixed with the malic acid/NaOH buffer (20 mM, pH 3.0, without additional salt) without IVT-mRNA under vortex mixing. The volume ratio of ethanol and buffer was 1:1, 1:3, and 1:7. The resultant suspension of mRNA-free LNPs in the mixture of alcohol and malic acid buffer was diluted by MES/NaOH buffer (20 mM, pH 6.0) at least 4-fold and transferred to Amicon Ultra-4-100K centrifugal units or Amicon Ultra-15-100K centrifugal units according to the preparation scale. The suspension of mRNA-free LNPs was centrifuged for concentration (1,000 × g, room temperature). The resultant mRNA-free LNPs that remained in the upper cassette were re-diluted with the MES/NaOH buffer at least 10-fold and centrifuged again (1,000 × g, room temperature). This buffer replacement was repeated twice. The resultant suspension of mRNA-free LNPs was collected and diluted to 40 mM total lipids.

Membrane emulsification method: The lipid stocks were prepared and mixed as described above. An AXF™ mini emulsification unit (Micropore technologies, Lazenby UK) was used for continuous membrane emulsification. The lipid mixture was mixed with the malic acid/NaOH buffer (20 mM, pH 3.0, without additional salt) without IVT-mRNA. Total flow rate varied from 15 mL/min to 60 mL/min. FRR (water/ethanol) varied 1:1 to 1:7. The resultant suspension of mRNA-free LNPs in the mixture of alcohol and malic acid buffer was diluted by MES/NaOH buffer (20 mM, pH 6.0) at least 4-fold and transferred to Amicon Ultra-4-100K centrifugal units or Amicon Ultra-15-100K centrifugal units according to the preparation scale. The suspension of mRNA-free LNPs was centrifuged for concentration (1000 ×g, room temperature). The resultant mRNA-free LNPs that remained in the upper cassette were re-diluted with the MES/NaOH buffer at least 10-fold and centrifuged again (1,000 × g, room temperature). This buffer replacement was repeated twice. The resultant suspension of mRNA-free LNPs was collected and diluted to 40 mM total lipids.

Evaluation of encapsulation efficiency

The encapsulation efficiency of the mRNA was evaluated via Ribogreen® assay. The samples were diluted to 1,000 ng/mL of mRNA with PBS(−). A calibration curve from 0 to 2,000 ng/mL of mRNA was prepared via sequential dilution. In other vessels, Ribogreen® reagent was diluted 200-fold in PBS with or without 0.4% w/v TritonX-100. An equal volume of the mRNA solution and the

Ribogreen® solution with or without TritonX-100 was mixed in the wells of a 96-well black plate (50 μL each). The plate was incubated for 5 min with shaking at 500 rpm in a shaking incubator. Fluorescence (Ex: 484, Em: 535) was evaluated using a plate reader (Infinite 200 PRO; Tecan, Switzerland). The recovery ratio was calculated as the ratio of total mRNA (with TritonX-100) to the mRNA input. Encapsulation efficiency was calculated as the ratio of the concentration of mRNA that was not encapsulated (without TritonX-100) to that of the total mRNA.

Electrophoresis

Tris-Borate-EDTA Buffer(10x) was 10-fold diluted with distilled water to prepare TBE buffer. One gram of Agarose S was suspended in 100 mL of TBE buffer and then heated to dissolve completely. The dissolved agarose gel solution was used for preparing 1% w/v non-denaturing agarose gel. SYBR™ Green II Nucleic Acid Gel Stain was diluted with the TBE buffer 5,000-fold to prepare a staining solution. mRNA encoding luciferase were mixed with mRNA-free LNPs to prepare mRNA-LNPs. The final concentration of mRNA was 100 μg/mL mRNA. The L/R ratio of the samples was 100 or 25. The particle properties of these samples were measured by dynamic light scattering and Ribogreen® assay. The samples for electrophoresis were prepared as follows.

	mRNA only	mRNA-LNPs only	mRNA-LNPs +pAsp	mRNA-LNPs +SDS
100 μg/mL mRNA solution	7 μL	-	-	-
100 μg/mL mRNA mRNA-LNP	-	7 μL	7 μL	7 μL
10 mg/mL pAsp	-	-	1 μL	1 μL
10% SDS	-	-	-	1 μL
Water	2 μL	2 μL	1 μL	-
Tris-Borate-EDTA Buffer(10x)	1 μL	1 μL	1 μL	1 μL

Before the electrophoresis, two μL of 50% glycerol/water was added to each sample to adjust the sample density. These samples were electrophoresed for 20 min at 100 V in TBE buffer. After the electrophoresis, the gel was stained with staining solution for 30 min. The stained gel was visualized by Gel Doc EZ gel imager (Biorad. CA. USA) using an UV tray. Exposure time was set as 0.5 sec. The intensity of bands was analyzed using Image Lab™ software equipped with a Gel Doc EZ gel imager. Relative band intensity was calculated by normalizing the band intensity by corresponding mRNA-LNPs + SDS samples.

Single-particle analysis

Nano-flow cytometry analysis was performed using a NanoFCM (NanoFCM Inc., Tokyo, Japan). The mRNA-LNPs were prepared using either the ethanol dilution or post-encapsulation methods with analysis via the NanoFCM. The mRNA-LNPs were labeled with DiD fluorescent dye so they could be detected on the PC5-A channel. mRNA molecules in each particle were stained with SYTO-9 nucleic acid staining reagent before measurement to detect them on the FITC-A channel. The side-scatter and fluorescence characteristics were simultaneously detected using single-photon counting modules. The laser power was consistent for all runs, with 5 mW for the 488 nm laser and 10 mW for the 638 nm laser. The sampling pressure was set to 1.0 kPa, and the lasers were aligned to maximize peak intensity in the side-scatter and fluorescence channels. The instrument was calibrated for particle concentration using 250 nm standard fluorescent silica beads (QC beads) and for particle sizing using a mixture of pre-mixed silica beads with diameters of 68, 91, 113, and 155 nm (S16M-Exo beads). The LNP samples were diluted with Tris-EDTA (TE) buffer (pH 8.0) to achieve an optimal range of 2,000 to 12,000 particles for 1 min at 1 kPa pressure. Each LNP that passed through the interrogation volume produced a pulse or burst of photocurrent on both the side-scatter and fluorescence detection channels. The integrated number of detected photons for each burst was recorded as the burst area. NanoFCM Profession 2.0 software was used to analyze the side-scatter burst area (SS-A) and fluorescence burst area (FL-A) signals of individual mRNA-LNP particles. Threshold values were determined with the auto function set to 'small signal'. Signals with DiD fluorescence were counted as LNPs. The mRNA(−) fraction and mRNA(+) fraction in the mRNA-LNPs formulation were distinguished based on the SYTO-9 fluorescence (Figure 2A, 2B). The size of the particles was calculated for the calibration by using QC beads (Figure 2D).

FRET cancellation analysis

FRET LNPs(RtoU/Liq) were prepared by labeling mRNA-free LNPs(RtoU/Liq) with 0.5 mol% DiA and 0.5 mol% DiD. These dyes were incorporated into the lipid mixture in ethanol. The particles were prepared as described above. The FRET LNPs(RtoU/Liq) were mixed with non-labelled mRNA-free LNPs(RtoU/Liq) at a ratio of 1:9. The mixture of the FRET LNPs(RtoU/Liq) and non-labelled mRNA-free LNPs(RtoU/Liq) was mixed with mRNA solution. After 5 minutes of incubation at 37 °C, the fluorescence of the DiA was measured using a plate reader (Infinite 200 PRO; Tecan, Switzerland). As a measure for FRET-cancelled control, mRNA-free LNPs(RtoU/Liq) with 0.05 mol% DiA and 0.05 mol% DiD were prepared. The FRET-cancellation was calculated as the DiA fluorescence relative to the FRET-cancelled control.

Cryo-TEM

A Cryo-TEM image was obtained using a JEM-2100F field-emission TEM apparatus (JEOL Co., Ltd., Tokyo, Japan) with an accelerating voltage of 120 kV [2]. For Cryo-TEM, 2 μL of the nanoparticle suspension (40 mM total lipids in Nuclease free water) was deposited onto a 200-mesh copper grid covered with carbon film (Nisshin EM Co., Ltd., Tokyo, Japan). After removing the excess liquid using filter paper, the sample was rapidly vitrified by immersion in liquid ethane using a Leica CPC cryo-preparation chamber (Leica Microsystems, Wetzlar, Germany). The grid with the vitrified thin film was placed on a sample holder that was maintained below −170 °C using liquid nitrogen. Images were recorded using a CCD camera.

Steady-state anisotropy measurements of DPH

The steady-state fluorescence anisotropy of 1,6-diphenyl-1,3,5-hexatriene (DPH) was measured using a FP-8300 spectrofluorometer equipped with FDP-223 polarizers and a CTU-100 temperature controller (JASCO, Tokyo, Japan). For the preparation of fluorescence-labeled LNPs(RtoU) containing 0.5 mol% DPH, a chloroform solution of DPH (30mM) was added to the lipid mixture. The samples were excited at 360 nm by vertically polarized or horizontally polarized light, and the vertically or horizontally polarized fluorescence at 430 nm from each excitation condition was measured. The G factor was defined as $G = I_{HV}/I_{HH}$, where I_{HV} and I_{HH} denote the fluorescent intensity of vertically polarized emissions and horizontally polarized emissions with horizontally polarized excitation, respectively. Steady-state anisotropy was calculated as anisotropy = $(I_{VV} - G \times I_{VH})/(I_{VV} + 2G \times I_{VH})$, where I_{VV} and I_{VH} denote the fluorescent intensity of vertically polarized emissions and horizontally polarized emissions with vertically polarized excitation, respectively.

Cell culture

HeLa cells were cultured in Dulbecco's Modified Eagles Medium (DMEM, high glucose) that contained 10 v/v% FBS and 100 U/mL of penicillin/Streptomycin. Cells were cultured in 10 cm dishes (Nunc™ EasYDish™, Thermo Fisher Scientific, Waltham, MA, U.S.). The cells were cultured under an atmosphere of 5% CO_2/air at 37 °C and were passed using 0.25 w/v% Trypsin in a 1 mM EDTA-4Na solution when they reached 80% confluence. The typical passage timing involved an interval of 2 days. The cell line was tested for mycoplasma contamination via the use of a MycoStrip™. No mycoplasma contamination was found.

TNS binding assay

The apparent pKa of the LNPs was measured by TNS assay [3]. Buffers with different pH values

were prepared; 20 mM citric acid/NaOH buffer (with 150 mM NaCl, pH 3.0, 3.5, 4.0, 4.5, 5.0, 5.5), 20 mM sodium dihydrogen phosphate/NaOH buffer (with 150 mM NaCl, pH 6.0, 6.4, 6.8, 7.2, 7.6, 8.0), and 20 mM Tris/HCl buffer (with 150 mM NaCl pH8.5, 9.0, 9.5, 10.0). 6-(p-Toluidino)-2-naphthalenesulfonic acid sodium salt (TNS) was dissolved at 0.6 mM in Nuclease free water and stored at 4 °C. The TNS solution (2 µL) and each of the buffers (186 µL) were mixed to prepare working solutions of TNS with different values of pH. In the well of a 96-well black plate, 12 µL of the LNP solution (0.5 mM total lipid) was mixed with the working solution of TNS. After shaking (400 rpm, 10 min), the fluorescence of TNS (Ex:321, Em:447) was measured. The apparent pKa of the surface was calculated as the pH at which the LNP showed 50% of the maximum fluorescence.

In vitro luciferase assay using an incubator type luminometer

HeLa cells (10 × 10^4 cells/2 mL) were seeded in 3.5 cm dishes (Nunc™ EasYDish™, Thermo Fisher Scientific, Waltham, MA, U.S.) 24 hours before transfection. The medium was replaced with fresh culture medium supplemented with 100 µM of D-luciferin potassium (Wako) and the mLuc-LNPs(RtoU) containing 400 ng of the mRNA. The cells were then placed in an incubation-type luminometer Kronos (ATTO, Tokyo, Japan) and the luciferase activity was measured for 2 min at 1 hour intervals.

In vivo luciferase assay using an In Vivo Imaging System (IVIS)

BALB/c mice (female, 6 weeks) were administered with the mRNA-LNPs containing luciferase mRNA. The dose of the mRNA was 0.1 mg/kg. At 6 hours after administration, the mice were intraperitoneally injected with D-luciferin potassium solution in PBS(−) (3 mg/200 µL/head). At 5 minutes after the intraperitoneal injection, the luminescence from both the mouse body and excised tissues (Liver, Heart, Spleen, Kidney, and Lung) was measured using the In Vivo Imaging System IVIS (Perkin Elmer Japan, Yokohama, Japan). Imaging parameters were as follows: Exposure time = 10 seconds, Binning = medium, F/stop = 1, Emission filter = open, and Subject height = 1.5 cm.

Antigen-Specific Total IgG Quantification

BALB/c mice were intramuscularly administered with mRNA-LNPs containing OVA mRNA or SARS-CoV-2-Spike mRNA at a dose of 1.5 µg/head. The sequences of the templates are summarized in the following sections. The administration was done three times in two-week intervals. Blood collection was carried out on days 14, 28, and 42. Blood serum was obtained by incubating the blood at room temperature (RT) for 2 h following centrifugation (4 °C, 2,000 ×g, 10 min). The antigen-specific antibody was quantified using an enzyme-linked immunosorbent assay

(ELISA). Plate wells (clear flat-bottomed immuno non-sterile 96-well plates, Thermo Scientific, Waltham, MA, USA) were coated either with 100 µL of OVA protein (10 µg/mL in 50 mM $NaHCO_3$ pH 9.6) and let stand for 16 h at 4 °C or with recombinant SARS-CoV-2 S1 RBD recombinant protein (Adipogen Life Sciences, Basel, Switzer-land). The plates were washed with 0.1% (w/v) Tween 20 in D-PBS (−) and then blocked with 5% (w/v) Fetal Bovine Serum (FBS, #10270, Gibco, New York, USA, Thermo Fisher Scientific, Waltham, Massachusetts, US) following incubation (37 °C, 2 h). Samples of blood serum were diluted with 0.1% (w/v) Tween 20 in D-PBS (−) and added into the wells at concentrations of 1/100 following incubation (RT, 1 h). The plates were washed, and a 1/2,000 dilution of Goat anti-Mouse IgG-Fc Fragment Antibody HRP Conjugated (A90-131P, Bethyl Laboratories, Montgomery, TX, USA) was added into the wells following incubation (RT, 1 h). The plates were once again washed, and 100 µL of TMB Solution (CL07-100ML, Merck Millipore, Burlington, MA, USA) was added into each well. The plates were incubated in a dark environment (RT, 30 min), and then 100 µL of 1 M H_2SO_4 (stop solution) was added. The absorbances were measured using a plate reader (Infinite M200 PRO, TECAN, Männedorf, Switzerland) set to λ = 450 nm. The antigen-specific total IgG levels were determined directly from the absorbance values.

S3. Sequence of pDNA template for vaccination

Ovalbumin

The sequence appears below:

TAATACGACTCACTATAGGGTCCCGCAGTCGGCGTCCAGCGGCTCTGCTTGTTCGTGTGTGTGTCGTTGCAGGCCTT
ATTCAAGCTTGAGGATGGGCTCTATCGGCGCTGCCAGCATGGAATTCTGCTTCGACGTGTTCAAAGAGCTGAAGGTC
CACCACGCCAACGAGAACATCTTCTACTGCCCTATCGCCATCATGAGCGCCCTGGCCATGGTGTACCTGGGCGCCA
AGGACAGCACCAGAACACAGATCAACAAGGTCGTCCGCTTCGACAAGCTGCCTGGCTTCGGCGATTCTATCGAGGC
CCAGTGTGGCACCTCTGTGAACGTGCACAGCAGCCTGAGAGACATCCTGAACCAGATCACCAAGCCTAACGACGTG
TACAGCTTCAGCCTGGCCAGCAGACTGTACGCCGAGGAAAGATACCCCATCCTGCCTGAGTACCTGCAGTGCGTGA
AAGAGCTGTACAGAGGCGGCCTGGAACCTATCAACTTCCAGACAGCCGCCGACCAGGCCAGAGAGCTGATCAATTC
TTGGGTCGAGAGCCAGACCAACGGCATCATCAGAAACGTGCTGCAGCCCAGCAGCGTGGACTCTCAGACAGCTATG
GTGCTGGTCAACGCCATCGTGTTTAAAGGCCTGTGGGAAAAGACCTTCAAGGACGAGGACACCCAGGCCATGCCTT
TCAGAGTGACCGAGCAAGAGTCCAAGCCTGTGCAGATGATGTACCAGATCGGCCTGTTTAGAGTGGCCTCCATGGC
CTCCGAGAAGATGAAGATCCTGGAACTGCCTTTCGCCAGCGGCACCATGTCTATGCTGGTGCTGCTGCCTGATGAG
GTGTCCGGACTGGAACAGCTGGAATCCATCATCAACTTCGAGAAGCTGACCGAGTGGACCAGCAGCAACGTGATGG
AAGAACGGAAGATCAAGGTCTACCTGCCTCGGATGAAGATGGAAGAGAAGTACAACCTGACCAGCGTGCTGATGGC
TATGGGCATCACCGATGTGTTCAGCAGCAGCGCCAACCTGAGCGGCATCTCTTCTGCCGAGAGCCTGAAGATCAGC
CAGGCCGTGCATGCTGCTCACGCCGAGATCAATGAGGCCGGCAGAGAAGTCGTGGGCTCTGCTGAAGCAGGCGTT
GACGCTGCCTCTGTGTCCGAGGAATTCAGAGCCGACCATCCTTTTCTGTTCTGCATCAAGCACATTGCCACCAACGC
CGTGCTGTTCTTCGGCAGATGTGTGTCCCCTTGAGACTAGTGCATCACATTTAAAAGCATCTCAGCCTACCATGAGAA
TAAGAGAAAGAAAATGAAGATCAATAGCTTATTCATCTCTTTTTCTTTTTCGTTGGTGTAAAGCCAACACCCTGTCTAAA
AAACATAAATTTCTTTAATCATTTTGCCTCTTTTCTCTGTGCTTCAATTAATAAAAAATGGAAAGAACCTAGATCTAAAAA
AAAAAAAAAAAAAAAAAAAAAAAAAATGAAGAGCCGTACGGGCGCGCCTAGGCGCGATTCCGCTTCCTCGCTCACTGA
CTCGCTGCGCTCGGTCGTTCGGCTGCGGCGAGCGGTATCAGCTCACTCAAAGGCGGTAATACGGTTATCCACAGAA
TCAGGGGATAACGCAGGAAAGAACATGTGAGCAAAAGGCCAGCAAAAGGCCAGGAACCGTAAAAAGGCCGCGTTGC
TGGCGTTTTTCCATAGGCTCCGCCCCCCTGACGAGCATCACAAAAATCGACGCTCAAGTCAGAGGTGGCGAAACCC
GACAGGACTATAAAGATACCAGGCGTTTCCCCCTGGAAGCTCCCTCGTGCGCTCTCCTGTTCCGACCCTGCCGCTTA
CCGGATACCTGTCCGCCTTTCTCCCTTCGGGAAGCGTGGCGCTTTCTCATAGCTCACGCTGTAGGTATCTCAGTTCG
GTGTAGGTCGTTCGCTCCAAGCTGGGCTGTGTGCACGAACCCCCCGTTCAGCCCGACCGCTGCGCCTTATCCGGTA
ACTATCGTCTTGAGTCCAACCCGGTAAGACACGACTTATCGCCACTGGCAGCAGCCACTGGTAACAGGATTAGCAGA
GCGAGGTATGTAGGCGGTGCTACAGAGTTCTTGAAGTGGTGGCCTAACTACGGCTACACTAGAAGAACAGTATTTGG
TATCTGCGCTCTGCTGAAGCCAGTTACCTTCGGAAAAAGAGTTGGTAGCTCTTGATCCGGCAAACAAACCACCGCTG
GTAGCGGTGGTTTTTTTGTTTGCAAGCAGCAGATTACGCGCAGAAAAAAAGGATCTCAAGAAGATCCTTTGATCTTTT
CTACGGGGTCTGACGCTCAGTGGAACGAAAACTCACGTTAAGGGATTTTGGTCATGAGATTATCAAAAAGGATCTTCA
CCTAGATCCTTTTAAATTAAAAATGAAGTTTTAAATCAATCTAAAGTATATATGAGTAAACTTGGTCTGACAGTTACCAA
TGCTTAATCAGTGAGGCACCTATCTCAGCGATCTGTCTATTTCGTTCATCCATAGTTGCCTGACTCCCCGTCGTGTAG
ATAACTACGATACGGGAGGGCTTACCATCTGGCCCCAGTGCTGCAATGATACCGCGAGATCCACGCTCACCGGCTC
CAGATTTATCAGCAATAAACCAGCCAGCCGGAAGGGCCGAGCGCAGAAGTGGTCCTGCAACTTTATCCGCCTCCATC
CAGTCTATTAATTGTTGCCGGGAAGCTAGAGTAAGTAGTTCGCCAGTTAATAGTTTGCGCAACGTTGTTGCCATTGCT
ACAGGCATCGTGGTGTCACGCTCGTCGTTTGGTATGGCTTCATTCAGCTCCGGTTCCCAACGATCAAGGCGAGTTAC
ATGATCCCCCATGTTGTGCAAAAAAGCGGTTAGCTCCTTCGGTCCTCCGATCGTTGTCAGAAGTAAGTTGGCCGCAG

TGTTATCACTCATGGTTATGGCAGCACTGCATAATTCTCTTACTGTCATGCCATCCGTAAGATGCTTTTCTGTGACTGG
TGAGTACTCAACCAAGTCATTCTGAGAATAGTGTATGCGGCGACCGAGTTGCTCTTGCCCGGCGTCAATACGGGATA
ATACCGCGCCACATAGCAGAACTTTAAAAGTGCTCATCATTGGAAAACGTTCTTCGGGGCGAAAACTCTCAAGGATCT
TACCGCTGTTGAGATCCAGTTCGATGTAACCCACTCGTGCACCCAACTGATCTTCAGCATCTTTTACTTTCACCAGCG
TTTCTGGGTGAGCAAAAACAGGAAGGCAAAATGCCGCAAAAAAGGGAATAAGGGCGACACGGAAATGTTGAATACTC
ATACTCTTCCTTTTTCAATATTATTGAAGCATTTATCAGGGTTATTGTCTCATGAGCGGATACATATTTGAATGTATTTA
GAAAAATAAACAAATAGGGGTTCCGCGCACATTTCCCCGAAAAGTGCCACCTGACGTCTAAGAAACCATTATTATCAT
GACATTAACCTATAAAAATAGGCGTATCACGAGGCCCTTTCGTC

S3. Sequence of pDNA template for vaccination

SARS-CoV-2-Spike

The sequence appears below:

TAATACGACTCACTATAGGGAGAATAAACTAGTATTCTTCTGGTCCCCACAGACTCAGAGAGAACCCGCCACCATGTT
CGTGTTCCTGGTGCTGCTGCCTCTGGTGTCCAGCCAGTGTGTGAACCTGACCACCAGAACACAGCTGCCTCCAGCC
TACACCAACAGCTTTACCAGAGGCGTGTACTACCCCGACAAGGTGTTCAGATCCAGCGTGCTGCACTCTACCCAGGA
CCTGTTCCTGCCTTTCTTCAGCAACGTGACCTGGTTCCACGCCATCCACGTGTCCGGCACCAATGGCACCAAGAGAT
TCGACAACCCCGTGCTGCCCTTCAACGACGGGGTGTACTTTGCCAGCACCGAGAAGTCCAACATCATCAGAGGCTG
GATCTTCGGCACCACACTGGACAGCAAGACCCAGAGCCTGCTGATCGTGAACAACGCCACCAACGTGGTCATCAAA
GTGTGCGAGTTCCAGTTCTGCAACGACCCCTTCCTGGGCGTCTACTACCACAAGAACAACAAGAGCTGGATGGAAAG
CGAGTTCCGGGTGTACAGCAGCGCCAACAACTGCACCTTCGAGTACGTGTCCCAGCCTTTCCTGATGGACCTGGAA
GGCAAGCAGGGCAACTTCAAGAACCTGCGCGAGTTCGTGTTTAAGAACATCGACGGCTACTTCAAGATCTACAGCAA
GCACACCCCTATCAACCTCGTGCGGGATCTGCCTCAGGGCTTCTCTGCTCTGGAACCCCTGGTGGATCTGCCCATC
GGCATCAACATCACCCGGTTTCAGACACTGCTGGCCCTGCACAGAAGCTACCTGACACCTGGCGATAGCAGCAGCG
GATGGACAGCTGGTGCCGCCGCTTACTATGTGGGCTACCTGCAGCCTAGAACCTTCCTGCTGAAGTACAACGAGAA
CGGCACCATCACCGACGCCGTGGATTGTGCTCTGGATCCTCTGAGCGAGACAAAGTGCACCCTGAAGTCCTTCACC
GTGGAAAAGGGCATCTACCAGACCAGCAACTTCCGGGTGCAGCCCACCGAATCCATCGTGCGGTTCCCCAATATCA
CCAATCTGTGCCCCTTCGGCGAGGTGTTCAATGCCACCAGATTCGCCTCTGTGTACGCCTGGAACCGGAAGCGGAT
CAGCAATTGCGTGGCCGACTACTCCGTGCTGTACAACTCCGCCAGCTTCAGCACCTTCAAGTGCTACGGCGTGTCCC
CTACCAAGCTGAACGACCTGTGCTTCACAAACGTGTACGCCGACAGCTTCGTGATCCGGGGAGATGAAGTGCGGCA
GATTGCCCCTGGACAGACAGGCAAGATCGCCGACTACAACTACAAGCTGCCCGACGACTTCACCGGCTGTGTGATT
GCCTGGAACAGCAACAACCTGGACTCCAAAGTCGGCGGCAACTACAATTACCTGTACCGGCTGTTCCGGAAGTCCAA
TCTGAAGCCCTTCGAGCGGGACATCTCCACCGAGATCTATCAGGCCGGCAGCACCCCTTGTAACGGCGTGGAAGGC
TTCAACTGCTACTTCCCACTGCAGTCCTACGGCTTTCAGCCCACAAATGGCGTGGGCTATCAGCCCTACAGAGTGGT
GGTGCTGAGCTTCGAACTGCTGCATGCCCCTGCCACAGTGTGCGGCCCTAAGAAAAGCACCAATCTCGTGAAGAAC
AAATGCGTGAACTTCAACTTCAACGGCCTGACCGGCACCGGCGTGCTGACAGAGAGCAACAAGAAGTTCCTGCCATT
CCAGCAGTTTGGCCGGGATATCGCCGATACCACAGACGCCGTTAGAGATCCCCAGACACTGGAAATCCTGGACATC
ACCCCTTGCAGCTTCGGCGGAGTGTCTGTGATCACCCCTGGCACCAACACCAGCAATCAGGTGGCAGTGCTGTACC
AGGACGTGAACTGTACCGAAGTGCCCGTGGCCATTCACGCCGATCAGCTGACACCTACATGGCGGGTGTACTCCAC
CGGCAGCAATGTGTTTCAGACCAGAGCCGGCTGTCTGATCGGAGCCGAGCACGTGAACAATAGCTACGAGTGCGAC
ATCCCCATCGGCGCTGGAATCTGCGCCAGCTACCAGACACAGACAAACAGCCCTCGGAGAGCCAGAAGCGTGGCCA
GCCAGAGCATCATTGCCTACACAATGTCTCTGGGCGCCGAGAACAGCGTGGCCTACTCCAACAACTCTATCGCTATC
CCCACCAACTTCACCATCAGCGTGACCACAGAGATCCTGCCTGTGTCCATGACCAAGACCAGCGTGGACTGCACCAT
GTACATCTGCGGCGATTCCACCGAGTGCTCCAACCTGCTGCTGCAGTACGGCAGCTTCTGCACCCAGCTGAATAGA
GCCCTGACAGGGATCGCCGTGGAACAGGACAAGAACACCCAAGAGGTGTTCGCCCAAGTGAAGCAGATCTACAAGA
CCCCTCCTATCAAGGACTTCGGCGGCTTCAATTTCAGCCAGATTCTGCCCGATCCTAGCAAGCCCAGCAAGCGGAGC
TTCATCGAGGACCTGCTGTTCAACAAAGTGACACTGGCCGACGCCGGCTTCATCAAGCAGTATGGCGATTGTCTGGG
CGACATTGCCGCCAGGGATCTGATTTGCGCCCAGAAGTTTAACGGACTGACAGTGCTGCCTCCTCTGCTGACCGATG
AGATGATCGCCCAGTACACATCTGCCCTGCTGGCCGGCACAATCACAAGCGGCTGGACATTTGGAGCAGGCGCCGC
TCTGCAGATCCCCTTTGCTATGCAGATGGCCTACCGGTTCAACGGCATCGGAGTGACCCAGAATGTGCTGTACGAGA
ACCAGAAGCTGATCGCCAACCAGTTCAACAGCGCCATCGGCAAGATCCAGGACAGCCTGAGCAGCACAGCAAGCGC

CCTGGGAAAGCTGCAGGACGTGGTCAACCAGAATGCCCAGGCACTGAACACCCTGGTCAAGCAGCTGTCCTCCAAC
TTCGGCGCCATCAGCTCTGTGCTGAACGATATCCTGAGCAGACTGGACCCTCCTGAGGCCGAGGTGCAGATCGACA
GACTGATCACAGGCAGACTGCAGAGCCTCCAGACATACGTGACCCAGCAGCTGATCAGAGCCGCCGAGATTAGAGC
CTCTGCCAATCTGGCCGCCACCAAGATGTCTGAGTGTGTGCTGGGCCAGAGCAAGAGAGTGGACTTTTGCGGCAAG
GGCTACCACCTGATGAGCTTCCCTCAGTCTGCCCCTCACGGCGTGGTGTTTCTGCACGTGACATATGTGCCCGCTCA
AGAGAAGAATTTCACCACCGCTCCAGCCATCTGCCACGACGGCAAAGCCCACTTTCCTAGAGAAGGCGTGTTCGTGT
CCAACGGCACCCATTGGTTCGTGACACAGCGGAACTTCTACGAGCCCCAGATCATCACCACCGACAACACCTTCGTG
TCTGGCAACTGCGACGTCGTGATCGGCATTGTGAACAATACCGTGTACGACCCTCTGCAGCCCGAGCTGGACAGCT
TCAAAGAGGAACTGGACAAGTACTTTAAGAACCACACAAGCCCCGACGTGGACCTGGGCGATATCAGCGGAATCAAT
GCCAGCGTCGTGAACATCCAGAAAGAGATCGACCGGCTGAACGAGGTGGCCAAGAATCTGAACGAGAGCCTGATCG
ACCTGCAAGAACTGGGGAAGTACGAGCAGTACATCAAGTGGCCCTGGTACATCTGGCTGGGCTTTATCGCCGGACT
GATTGCCATCGTGATGGTCACAATCATGCTGTGTTGCATGACCAGCTGCTGTAGCTGCCTGAAGGGCTGTTGTAGCT
GTGGCAGCTGCTGCAAGTTCGACGAGGACGATTCTGAGCCCGTGCTGAAGGGCGTGAAACTGCACTACACATGATG
ACTCGAGCTGGTACTGCATGCACGCAATGCTAGCTGCCCCTTTCCCGTCCTGGGTACCCCGAGTCTCCCCCGACCT
CGGGTCCCAGGTATGCTCCCACCTCCACCTGCCCCACTCACCACCTCTGCTAGTTCCAGACACCTCCCAAGCACGCA
GCAATGCAGCTCAAAACGCTTAGCCTAGCCACACCCCCACGGGAAACAGCAGTGATTAACCTTTAGCAATAAACGAA
AGTTTAACTAAGCTATACTAACCCCAGGGTTGGTCAATTTCGTGCCAGCCACACCCTGGAGCTAGCAAAAAAAAAAAA
AAAAAAAAAAAAAAAAAATGAAGAGCCGTACGGGCGCGCCTAGGCGCGATTCCGCTTCCTCGCTCACTGACTCGCTG
CGCTCGGTCGTTCGGCTGCGGCGAGCGGTATCAGCTCACTCAAAGGCGGTAATACGGTTATCCACAGAATCAGGGG
ATAACGCAGGAAAGAACATGTGAGCAAAAGGCCAGCAAAAGGCCAGGAACCGTAAAAAGGCCGCGTTGCTGGCGTT
TTTCCATAGGCTCCGCCCCCCTGACGAGCATCACAAAAATCGACGCTCAAGTCAGAGGTGGCGAAACCCGACAGGA
CTATAAAGATACCAGGCGTTTCCCCCTGGAAGCTCCCTCGTGCGCTCTCCTGTTCCGACCCTGCCGCTTACCGGATA
CCTGTCCGCCTTTCTCCCTTCGGGAAGCGTGGCGCTTTCTCATAGCTCACGCTGTAGGTATCTCAGTTCGGTGTAGG
TCGTTCGCTCCAAGCTGGGCTGTGTGCACGAACCCCCCGTTCAGCCCGACCGCTGCGCCTTATCCGGTAACTATCG
TCTTGAGTCCAACCCGGTAAGACACGACTTATCGCCACTGGCAGCAGCCACTGGTAACAGGATTAGCAGAGCGAGG
TATGTAGGCGGTGCTACAGAGTTCTTGAAGTGGTGGCCTAACTACGGCTACACTAGAAGAACAGTATTTGGTATCTG
CGCTCTGCTGAAGCCAGTTACCTTCGGAAAAAGAGTTGGTAGCTCTTGATCCGGCAAACAAACCACCGCTGGTAGCG
GTGGTTTTTTTGTTTGCAAGCAGCAGATTACGCGCAGAAAAAAAGGATCTCAAGAAGATCCTTTGATCTTTTCTACGG
GGTCTGACGCTCAGTGGAACGAAAACTCACGTTAAGGGATTTTGGTCATGAGATTATCAAAAAGGATCTTCACCTAGA
TCCTTTTAAATTAAAAATGAAGTTTTAAATCAATCTAAAGTATATATGAGTAAACTTGGTCTGACAGTTACCAATGCTTA
ATCAGTGAGGCACCTATCTCAGCGATCTGTCTATTTCGTTCATCCATAGTTGCCTGACTCCCCGTCGTGTAGATAACT
ACGATACGGGAGGGCTTACCATCTGGCCCCAGTGCTGCAATGATACCGCGAGATCCACGCTCACCGGCTCCAGATT
TATCAGCAATAAACCAGCCAGCCGGAAGGGCCGAGCGCAGAAGTGGTCCTGCAACTTTATCCGCCTCCATCCAGTCT
ATTAATTGTTGCCGGGAAGCTAGAGTAAGTAGTTCGCCAGTTAATAGTTTGCGCAACGTTGTTGCCATTGCTACAGGC
ATCGTGGTGTCACGCTCGTCGTTTGGTATGGCTTCATTCAGCTCCGGTTCCCAACGATCAAGGCGAGTTACATGATC
CCCCATGTTGTGCAAAAAAGCGGTTAGCTCCTTCGGTCCTCCGATCGTTGTCAGAAGTAAGTTGGCCGCAGTGTTAT
CACTCATGGTTATGGCAGCACTGCATAATTCTCTTACTGTCATGCCATCCGTAAGATGCTTTTCTGTGACTGGTGAGT
ACTCAACCAAGTCATTCTGAGAATAGTGTATGCGGCGACCGAGTTGCTCTTGCCCGGCGTCAATACGGGATAATACC
GCGCCACATAGCAGAACTTTAAAAGTGCTCATCATTGGAAAACGTTCTTCGGGGCGAAAACTCTCAAGGATCTTACCG
CTGTTGAGATCCAGTTCGATGTAACCCACTCGTGCACCCAACTGATCTTCAGCATCTTTTACTTTCACCAGCGTTTCTG
GGTGAGCAAAAACAGGAAGGCAAAATGCCGCAAAAAAGGGAATAAGGGCGACACGGAAATGTTGAATACTCATACTC
TTCCTTTTTCAATATTATTGAAGCATTTATCAGGGTTATTGTCTCATGAGCGGATACATATTTGAATGTATTTAGAAAAA
TAAACAAATAGGGGTTCCGCGCACATTTCCCCGAAAAGTGCCACCTGACGTCTAAGAAACCATTATTATCATGACATT
AACCTATAAAAATAGGCGTATCACGAGGCCCTTTCGTC

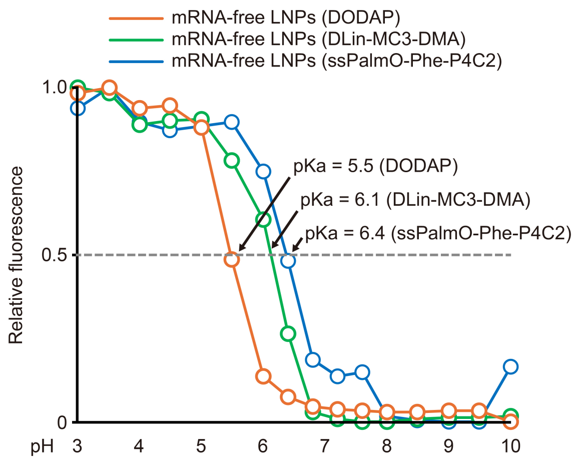

Figure S1. An apparent pKa of mRNA-free LNPs. The lipid composition for DODAP and DLin-MC3-DMA was ionizable lipid/DSPC/cholesterol/DMG-PEG2000 = 50/10/38.5/1.5 in molar ratio. The lipid composition for ssPalmO-Phe-P4C2 was ionizable lipid/DOPC/cholesterol/DMG-PEG2000 = 52.5/7.5/40/1.5 in molar ratio. An apparent pKa of the mRNA-free LNPs(RtoU/Liq) containing DODAP, DLin-MC3-DMA, or ssPalmO-Phe-P4C2 was evaluated via 6-(p-toluidino)-2-naphthalenesulfonate (TNS) binding assay [3]. The pKa was calculated as a pH at which fluorescence showed half of the maximum. As a result, it was indicated that the apparent pKa of the LNPs containing DODAP, DLin-MC3-DMA, or ssPalmO-Phe-P4C2 was 5.5, 6.1, and 6.4, respectively.

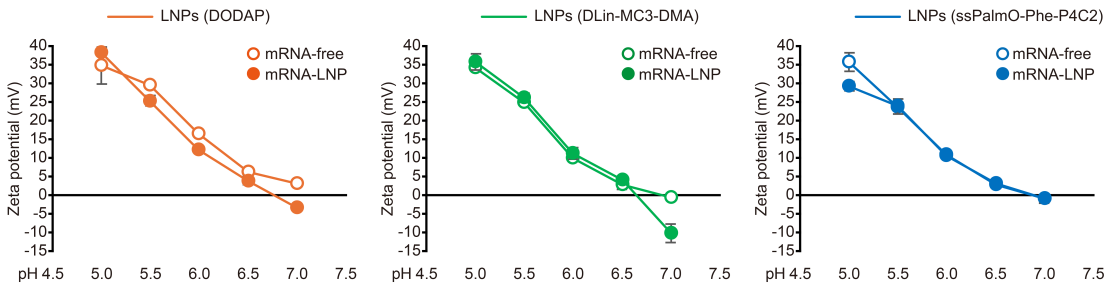

Figure S2. Zeta potential of LNPs at each pH before and after mRNA addition. Zeta potential of LNPs(RtoU) containing DODAP, DLin-MC3-DMA, and ssPalmO-Phe-P4C2 was evaluated with dynamic light scattering. The lipid composition for DODAP and DLin-MC3-DMA was ionizable lipid/DSPC/cholesterol/DMG-PEG2000 = 50/10/38.5/1.5 in molar ratio. The lipid composition for ssPalmO-Phe-P4C2 was ionizable lipid/DOPC/cholesterol/DMG-PEG2000 = 52.5/7.5/40/1.5 in molar ratio. The zeta potential of the particles at each pH indicated were evaluated before and after the mRNA addition, shown as mRNA-free and mRNA-LNP, respectively. As a result, the pH-dependent cationic charges of the mRNA-free LNPs were confirmed. Although the pKa of these mRNA-free LNPs was different (Figure S1), the zeta potential behavior was similar. Before mRNA addition, the zeta potential ranged from approximately 0 mV at pH 7.0 to +35 mV at pH 5.0. After mRNA addition, the zeta potential ranged from approximately −15 mV at pH 7.0 to + 40 mV at pH 5.0.

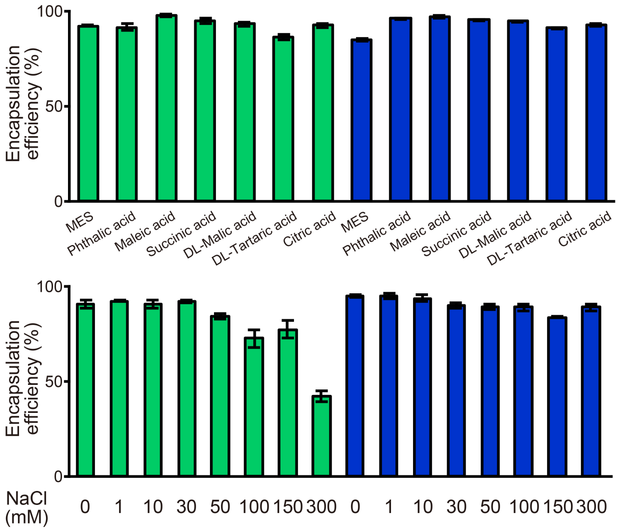

Figure S3. Effects of buffer components. mRNA-free LNPs(RtoU/Liq) containing DLin-MC3-DMA (green bars) or ssPalmO-Phe-P4C2 (blue bars) were prepared. The lipid compositions were DLin-MC3-DMA/DSPC/cholesterol/DMG-PEG2000 = 50/10/38.5/1.5 and ssPalmO-Phe-P4C2/DOPC/cholesterol/DMG-PEG2000 = 52.5/7.5/40/1.5. (Upper) mRNA-free LNPs(RtoU/Liq) containing DLin-MC3-DMA or ssPalmO-Phe-P4C2 were prepared with the various buffers indicated. The concentration of the buffers was 20 mM. The buffers were adjusted to pH 5.0 via NaOH solution. The mRNA-free LNPs(RtoU/Liq) were mixed with the mRNA in the same buffer to perform the post-encapsulation. As a result, the kind of buffers tested in this study did not affect the encapsulation efficiency. (Lower) mRNA-free LNPs(Rtou/Liq) containing DLin-MC3-DMA or ssPalmO-Phe-P4C2 were prepared with 20 mM of pH 5.0 MES buffer containing various NaCl concentrations, as indicated. The mRNA-free LNPs(RtoU/Liq) were mixed with mRNA in the same buffer to perform the post-encapsulation. As a result, the increase in the ionic strength with NaCl tended to slightly decrease the encapsulation efficiency.

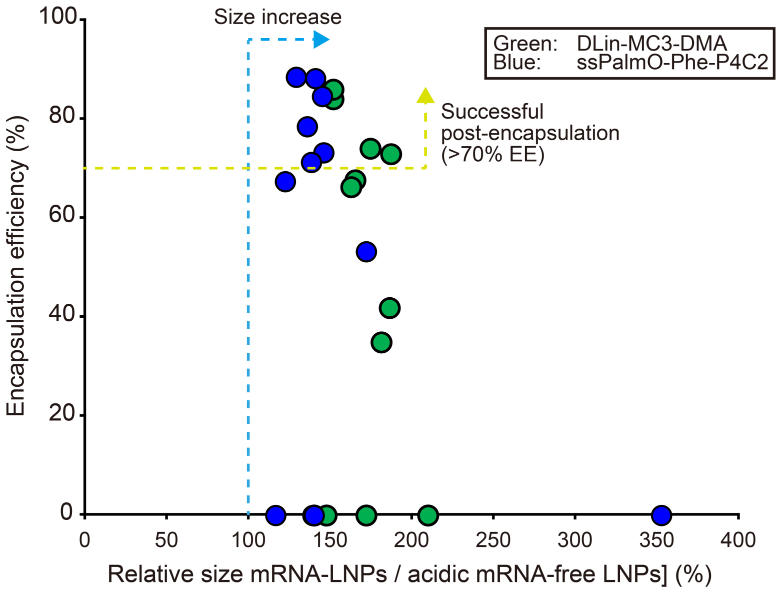

Figure S4. Correlation between size increase and encapsulation efficiency. The encapsulation efficiency after the post-encapsulation was plotted against the relative size increase of the LNPs. Green filled circles and blue filled circles show the results of LNPs containing DLin-MC3-DMA and ssPalmO-Phe-P4C2, respectively. The lipid compositions were DLin-MC3-DMA/DSPC/cholesterol/DMG-PEG2000 = 50/10/38.5/1.5 and ssPalmO-Phe-P4C2/DOPC/cholesterol/DMG-PEG2000 =52.5/7.5/40/1.5. The properties of the particles are shown in Figures 1F and 1G in the Main text. The values are listed in Tables S1 and S2, respectively. All particles showed size increase with the addition of mRNA. On the other hand, there are no clear trends in the relationship between the extent of relative size increase and encapsulation efficiency when the LNPs with different compositions were compared. The Pearson correlation coefficient was $R = -0.29$, which indicates only a weak negative correlation.

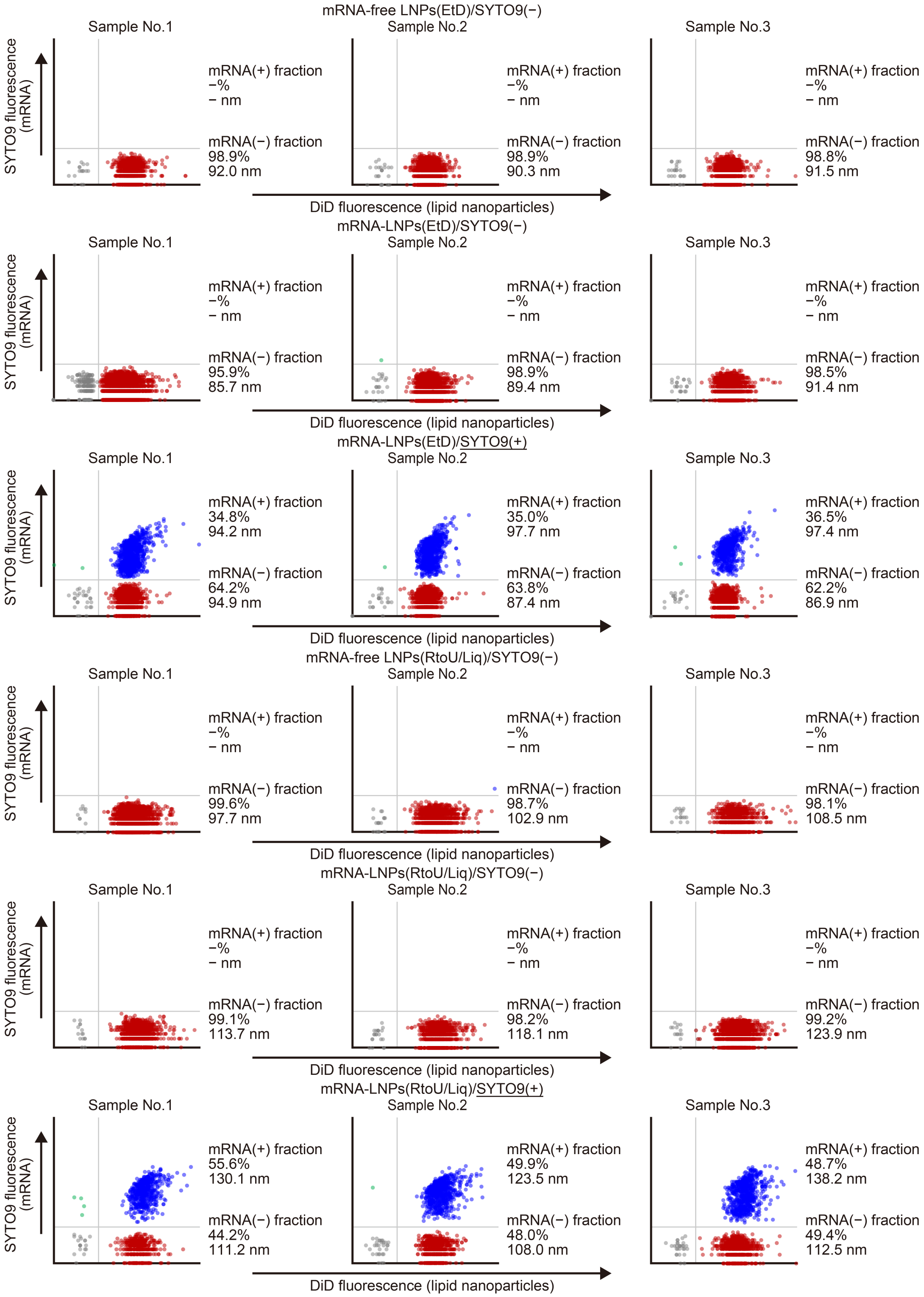

Figure S5. Additional dot plots of mRNA-LNPs(EtD) and mRNA-LNPs(RtoU/Liq). The plots of each single-particle analysis measurement were shown.

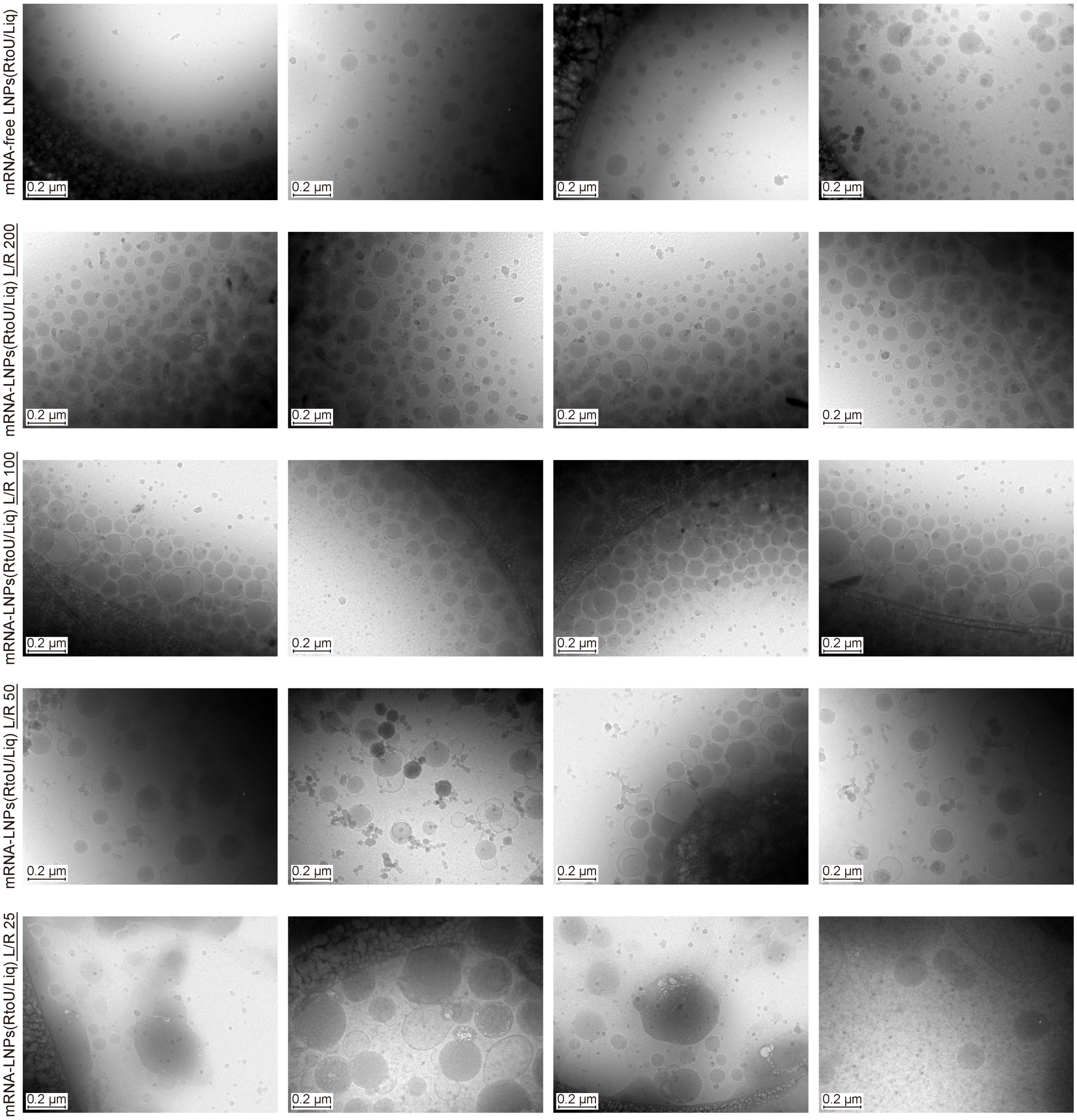

Figure S6. Cryo-TEM images. Cryo-TEM images of the mRNA-LNPs(RtoU/Liq) with L/R ranging from 200 to 25 as well as mRNA-free LNPs(RtoU/Liq) are shown. As the L/R ratio decreased, which corresponds to the increase in the mRNA amount, the size of the particles increased. Large non-spherical aggregates appeared at L/R 25. The LNPs prepared in this study had bleb-like structures on them [4]. These bleb-like structures were observed on the mRNA-free LNPs and on the mRNA-LNPs prepared by other preparation methods [2], which suggests that their formation could be due to the composition of the LNPs used and unrelated to the manufacturing methods used in the current study.

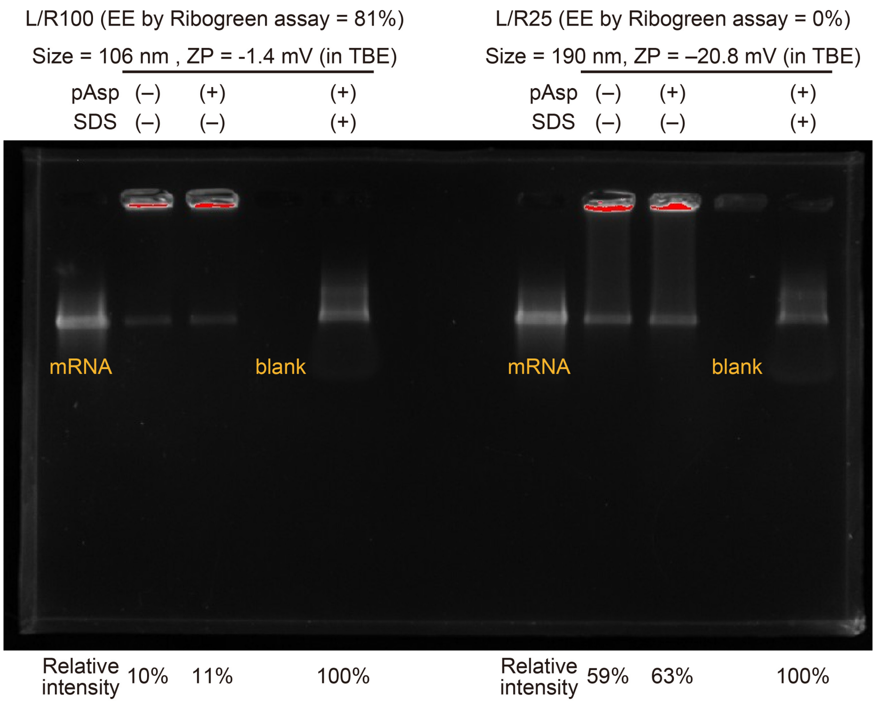

Figure S7. Electrophoresis of mRNA formulated at different L/R ratio. Electrophoresis experiments to distinguish free mRNA from complexed mRNA was conducted. The electrophoresis was performed in 1% w/v non-denaturing agarose gel in TBE buffer. The band intensity of mRNA-LNP samples were treated with the polyanion poly-aspartate (pAsp) to release the nucleic acids that were interacting electrostatically, and mRNA-LNP samples were treated with the surfactant sodium dodecyl sulfate (SDS) to completely dissolve the particles. In the L/R100 sample, which has an encapsulation efficiency of 81% as determined using Ribogreen® assay, only 11% of the total nucleic acids contained in the mRNA-LNP sample were detected as free mRNA. In the L/R25 sample, which has an encapsulation efficiency of 0% as determined using Ribogreen assay, 63% of the total nucleic acids were detected as free mRNA. This result suggests that a large portion of the unencapsulated mRNA are present as free mRNA at low L/R ratios. The residual 37% is released after particle dissolution with SDS treatment. EE; encapsulation efficiency.

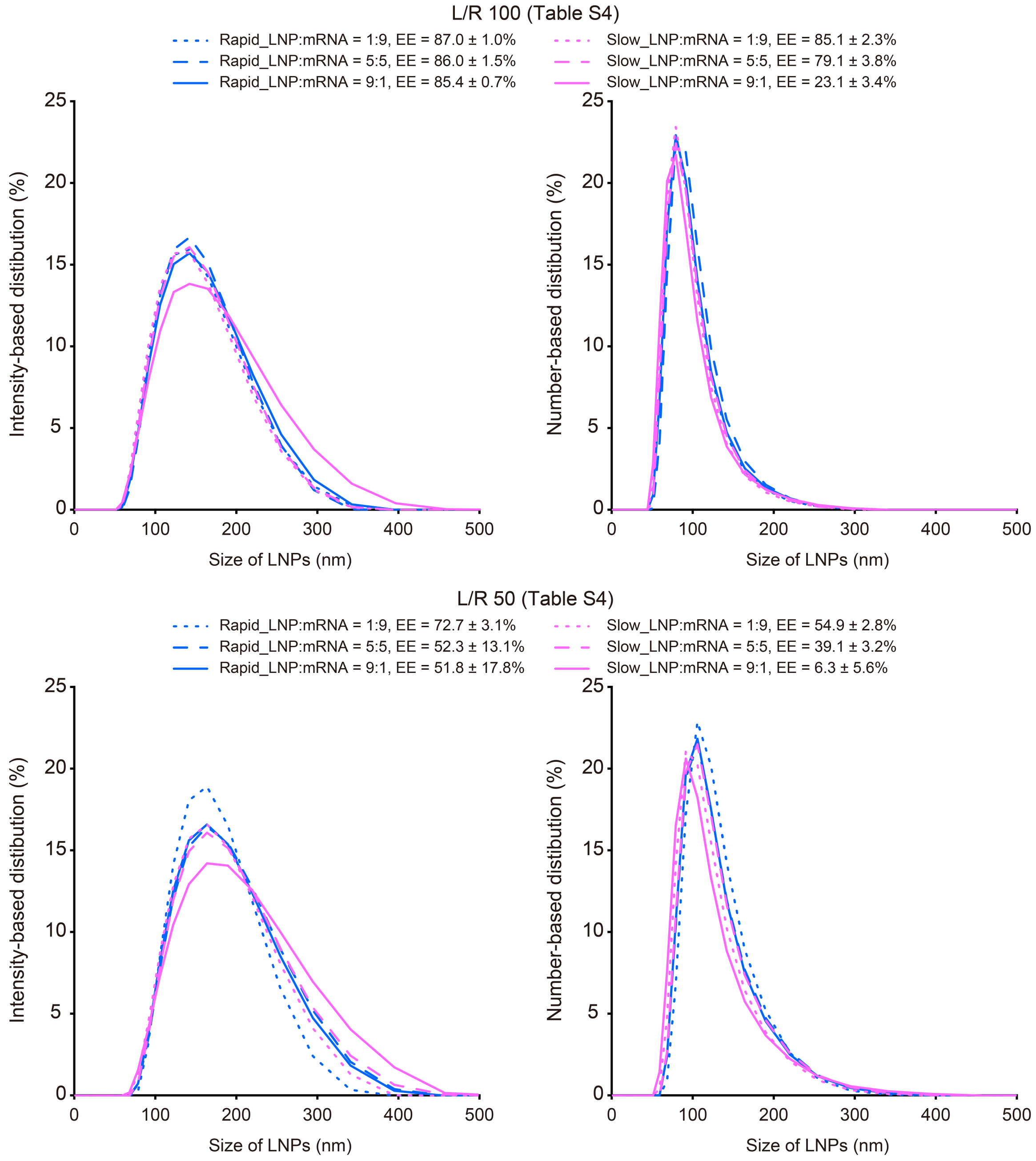

Figure S8. Size distribution of particles with different mixing procedures. Size distribution of the mRNA-LNPs(RtoU/Liq) are shown. EE means encapsulation efficiency of the mRNA. The samples in the same graph have the same final formulation composition, while the mixing method and volume ratio of the mRNA-free LNPs suspension and mRNA solution were different among them. Particle properties are shown in Table S4. Intensity-based size distribution and number-based size distribution are shown. In the case of both L/R 100 and L/R 50, the presence of larger particles with small numbers were suggested in the samples with lower encapsulation efficiency.

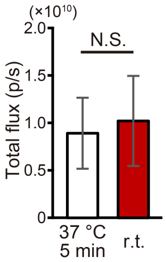

Figure S9. Effect of 37 °C incubation on the *in vivo* gene expression. In the preparation of mRNA-LNPs shown in the Main text, the mixture of the mRNA-free LNPs and mRNA was incubated at 37 °C for 5 minutes. This step was inserted into the overall protocol to exclude the impact of room temperature on the deviation of the results. In this experiment, the luciferase activity of the mRNA-LNPs(RtoU/Liq) on the liver after intravenous injection was obtained with or without the 37 °C incubation. As a result, the gene expression efficiency was comparable. This result indicated that the incubation at 37 °C was not actually necessary. Therefore, it was concluded that the mRNA-free LNPs(RtoU/Liq) could be used without heating.

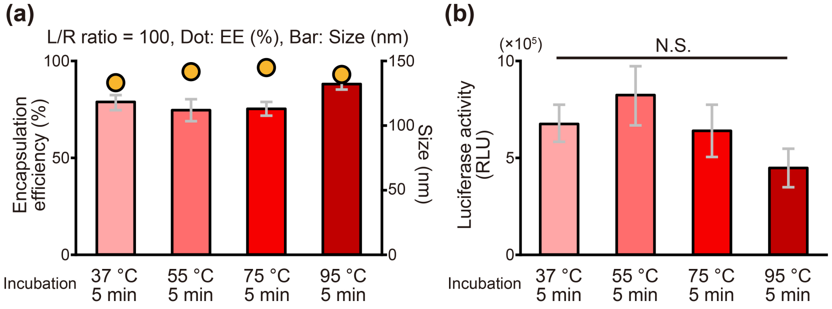

Figure S10. Effect of incubation temperature on *in vitro* gene expression. In the case of the previous Ready-to-Use formulation based on freeze-drying, incubation at high temperature over 75 °C was required to complete the post-encapsulation. The heating was particularly important for *in vitro* gene expression [2]. On the other hand, in the case of mRNA-free LNPs(RtoU/Liq), encapsulation efficiency and gene expression were not affected by the temperature of incubation. Therefore, mRNA-free LNPs(RtoU/Liq) could be used in both *in vivo* and *in vitro* situations without heating. These results again confirmed that the post-encapsulation method based on the mRNA-free LNPs(RtoU/Liq) has little effect on the mRNA quality.

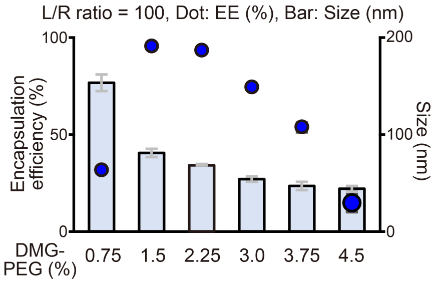

Figure S11. Effects of DMG-PEG2000 amount on post-encapsulation. The mRNA-free LNPs(RtoU) with the composition of ssPalmO-Phe-P4C2/DOPC/cholesterol = 52.5/7.5/40 was prepared with different amounts of DMG-PEG2000. After the post-encapsulation, the encapsulation efficiency was evaluated by Ribogreen® assay. At 1.5-3% of DMG-PEG2000, the encapsulation efficiency was more than 70%. On the other hand, the DMG-PEG2000 higher than 3.75% decreased the encapsulation efficiency. This decrease was probably due to the steric inhibition of high-surface DMG-PEG2000. On the other hand, the encapsulation efficiency decreased also in the case of 0.75% DMG-PEG2000. This decrease in the encapsulation efficiency could be explained from the viewpoint of size-dependency described in Figure S13.

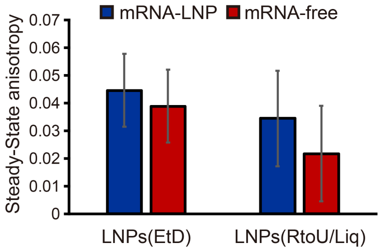

Figure S12. Steady-state anisotropy measurement. The fluidity of the hydrophobic chains was evaluated by measuring the steady-state anisotropy (rs) of the DPH. The rs values of DPH in phospholipids in gel phase were approximately 0.25-0.40, while the rs value of DPH in phospholipids in liquid crystalline phase was below 0.10 [5–7]. It should be pointed out that the rs values of the freeze-dried Ready-to-Use formulation was 0.12-0.15, and it decreased to below 0.05 by heating in the presence of mRNA [2]. The rs value of the LNPs(EtD) indicates that the lipid molecules in the LNPs have high fluidity. Since the rs value of LNPs(RtoU/Liq) was comparable to that of LNPs(EtD), fluidity of the LNPs(RtoU/Liq) was also high. This high fluidity due to omitting the freeze-drying process is one of the keys for successful post-encapsulation without additional heating.

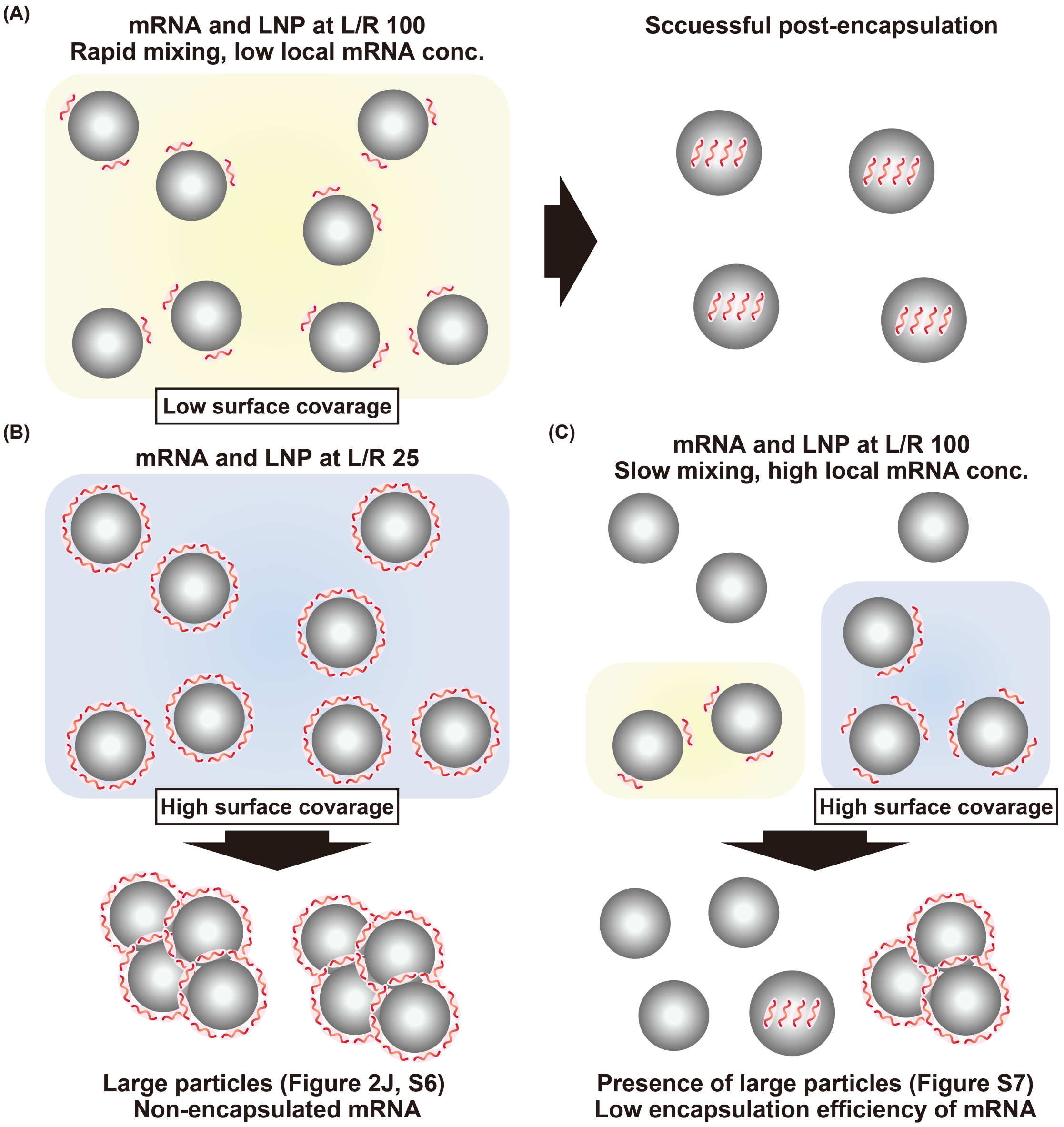

Figure S13. Hypothesis for the unsuccessful post-encapsulation. (A) By mixing a solution of low-concentration mRNA with a high-concentration of mRNA-free LNPs, mRNA could be encapsulated efficiently. Since the post-encapsulation requires LNP-to-LNP interaction, the LNP-rich conditions would be advantageous. (B) When the amount of mRNA was increased, encapsulation efficiency decreased with an increase in particle size. The high amount of mRNA might inhibit the migration of mRNA inside probably due to the segregation of lipids, and eventually form a large complex. (C) When a fraction of LNPs and mRNA are encountered under mRNA-rich conditions, the encapsulation efficiency also decreased, and a small number of large particles was observed. Therefore, even if it occurs locally, high surface coverage would have a negative impact on post-encapsulation. Further study is needed to reveal the properties of the large complexes.

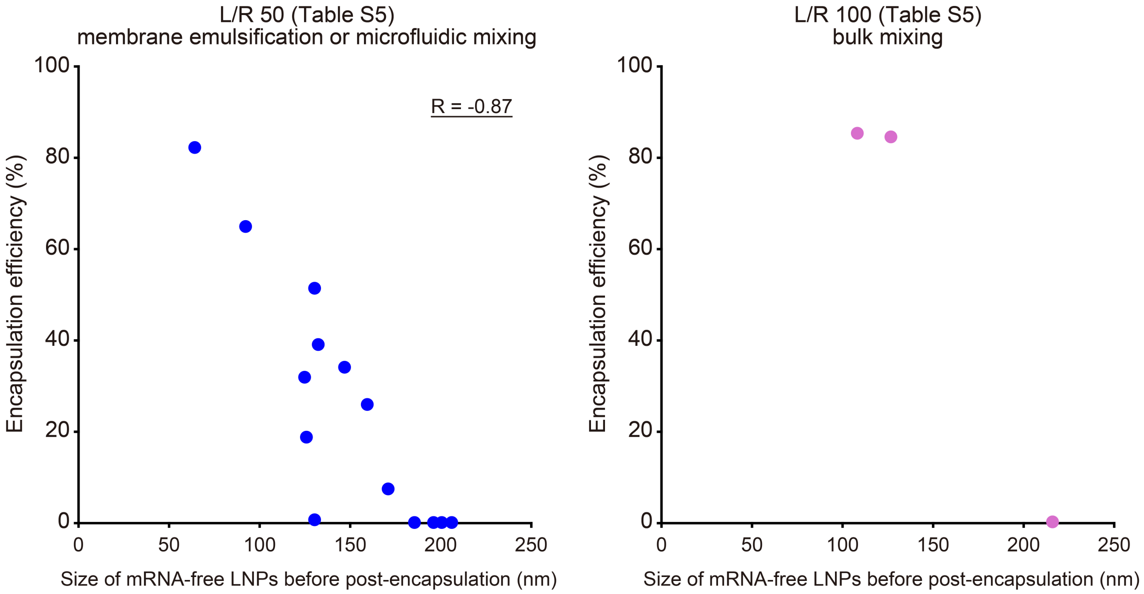

Figure S14. Size dependency of post-encapsulation. mRNA-free LNPs were prepared using microfluidic mixing (LNPs(MF)), membrane emulsification (LNPs(ME)), or bulk mixing (LNPs(BM)). By varying the buffer/ethanol ratio and total flow rate in the particle-formation process, different-sized mRNA-free LNPs were prepared with the same composition. The preparation conditions and particle properties are summarized in Table S5. In the case of LNPs(MF) and LNPs(ME), the L/R ratio was set to 50. In the case of LNPs(BM), the L/R ratio was set to 100. The encapsulation efficiency was plotted against the size of mRNA-free LNPs before mRNA addition. As a result, the encapsulation efficiency showed a strong negative correlation to the size of mRNA-free LNPs. The Pearson correlation coefficient was $R = -0.87$ for L/R 50 samples. Therefore, to perform the post-encapsulation successfully, down-sizing of the mRNA-free LNPs would be important.

This observation paradoxically suggests again that the high surface coverage of LNPs by mRNA has a negative impact on the post-encapsulation process. One possibility is the effect of reduced mobility of lipid molecules interacting with mRNA as described in the Main text. Another possibility is that the structural properties of mRNA adsorbed on the particles changed depending on its amount. When polyelectrolyte molecules adsorb on the surface of colloidal particles, the conformation of the polyelectrolyte changes according to their amount [8,9]. A relationship between the conformation and adsorption amount has been investigated [10]. In that report, the orientation of phosphate groups in nucleic acids changed across the point where the cationic charge of the lipid membrane is neutralized by phosphate groups. It was also reported that the mode of structural rearrangement of lipoplexes was affected by the amount of adsorbed nucleic acids [11,12]. Therefore, it is possible that the surface density of mRNA eventually determines whether or not the mRNA will be encapsulated via LNP-to-LNP fusion.

Table S1. Particle properties of DLin-MC3-DMA LNPs.

DLin-MC3 -DMA ratio (%)	DSPC ratio (%)	Cholesterol ratio (%)	Size of acidic fLNPs (nm) a)	Size of neutral fLNPs (nm) a)	Size of mRNA-LNPs (nm) a)	Encapsulation efficiency (%) b)	ZP of acidic fLNPs (mV) a)	ZP of neutral fLNPs (mV) a)	ZP of mRNA-LNPs (mV) a)	PdI of acidic fLNPs a)	PdI of neutral fLNPs a)	PdI of mRNA-LNPs a)
98.5	0	0	81.8 ± 4.2	79.5 ± 9.4	114.0 ± 12.2	0	21.4 ± 1.9	11.1 ± 0.9	−11.8 ± 2.0	0.09 ± 0.02	0.09 ± 0.02	0.07 ± 0.04
80	0	18.5	77.1 ± 6.7	79.3 ± 1.0	113.1 ± 7.4	0	17.2 ± 2.0	4.3 ± 1.6	−8.6 ± 0.9	0.14 ± 0.01	0.13 ± 0.01	0.10 ± 0.03
60	0	38.5	56.7 ± 11.6	61.6 ± 5.4	98.7 ± 5.4	73.8 ± 1.8	11.4 ± 0.8	−1.5 ± 0.4	−6.2 ± 1.5	0.17 ± 0.02	0.15 ± 0.01	0.05 ± 0.02
40	0	58.5	54.4 ± 5.1	52.6 ± 6.5	98.2 ± 43.8	34.9 ± 3.4	11.9 ± 0.02	−0.5 ± 0.6	−24.7 ± 0.6	0.24 ± 0.02	0.23 ± 0.02	0.23 ± 0.02
88.5	10	0	55.1 ± 7.2	43.8 ± 5.1	94.6 ± 4.3	0	18.7 ± 1.7	6.6 ± 2.2	−5.4 ± 0.6	0.20 ± 0.01	0.25 ± 0.05	0.14 ± 0.01
70	10	18.5	43.2 ± 1.0	28.6 ± 25.1	80.3 ± 2.1	41.7 ± 2.9	17.2 ± 1.0	2.2 ± 0.3	−2.5 ± 0.9	0.27 ± 0.01	0.30 ± 0.03	0.16 ± 0.004
50	10	38.5	43.6 ± 0.4	42.4 ± 0.9	65.9 ± 3.0	84.0 ± 2.0	10.5 ± 10.2	1.1 ± 0.4	0.4 ± 0.4	0.37 ± 0.01	0.34 ± 0.04	0.18 ± 0.01
30	10	58.5	42.4 ± 0.6	40.4 ± 4.3	64.1 ± 4.7	86.0 ± 1.5	14.7 ± 1.1	0.6 ± 0.4	−0.2 ± 0.1	0.43 ± 0.03	0.48 ± 0.07	0.24 ± 0.01
78.5	20	0	38.4 ± 4.3	44.0 ± 4.6	80.4 ± 2.4	0	17.2 ± 2.4	2.9 ± 4.0	−4.3 ± 1.1	0.27 ± 0.01	0.33 ± 0.04	0.16 ± 0.01
60	20	18.5	38.0 ± 5.3	39.8 ± 3.8	70.8 ± 1.1	72.8 ± 2.0	17.2 ± 1.2	2.2 ± 0.3	−1.4 ± 1.3	0.27 ± 0.01	0.27 ± 0.002	0.20 ± 0.01
40	20	38.5	36.4 ± 7.1	41.0 ± 1.9	60.0 ± 2.6	67.5 ± 5.9	15.3 ± 0.9	0.7 ± 0.7	−0.2 ± 0.3	0.45 ± 0.01	0.38 ± 0.02	0.29 ± 0.03
20	20	58.5	46.5 ± 1.0	43.7 ± 3.4	75.5 ± 8.9	66.0 ± 2.3	14.5 ± 1.0	2.2 ± 0.3	−1.2 ± 1.5	0.39 ± 0.02	0.39 ± 0.01	0.24 ± 0.01

(a) Size, PdI, and zeta potential (ZP) were measured by dynamic light scattering. Dynamic light scattering was performed in HEPES buffer. (b) Encapsulation efficiency was evaluated via Ribogreen® assay. The mean ± SD of three independent samples is shown. fLNPs; mRNA-free LNPs

Table S2. Particle properties of ssPalmO-Phe-P4C2 LNPs.

ssPalmO-Phe-P4C2 ratio (%)	DOPC ratio (%)	Cholesterol ratio (%)	Size of acidic fLNPs (nm) a)	Size of neutral fLNPs (nm) a)	Size of mRNA-LNPs (nm) a)	Encapsulation efficiency (%) b)	ZP of acidic fLNPs (mV) a)	ZP of neutral fLNPs (mV) a)	ZP of mRNA-LNPs (mV) a)	PdI of acidic fLNPs a)	PdI of neutral fLNPs a)	PdI of mRNA-LNPs a)
100	0	0	127.6 ± 8.7	130.3 ± 5.5	176.5 ± 4.7	0	19.1 ± 0.7	7.2 ± 1.0	−12.1 ± 3.1	0.09 ± 0.04	0.10 ± 0.06	0.05 ± 0.03
80	0	20	138.5 ± 3.4	136.8 ± 3.3	160.5 ± 2.3	0	11.5 ± 1.2	0.5 ± 0.1	−3.1 ± 0.4	0.07 ± 0.02	0.07 ± 0.01	0.06 ± 0.03
60	0	40	101.6 ± 4.9	103.5 ± 5.2	147.5 ± 5.6	73.2 ± 1.3	6.4 ± 3.9	−2.1 ± 0.5	−6.1 ± 3.1	0.06 ± 0.02	0.06 ± 0.03	0.02 ± 0.02
40	0	60	89.3 ± 5.5	90.9 ± 6.9	153.0 ± 6.4	53.2 ± 0.9	7.4 ± 0.9	−3.8 ± 0.8	−5.9 ± 2.6	0.13 ± 0.01	0.11 ± 0.03	0.11 ± 0.01
92.5	7.5	0	93.4 ± 2.4	92.3 ± 3.7	130.0 ± 3.1	0	24.3 ± 2.0	12.4 ± 0.5	5.1 ± 0.2	0.12 ± 0.01	0.10 ± 0.004	0.04 ± 0.01
72.5	7.5	20	87.8 ± 3.5	90.0 ± 4.4	121.0 ± 3.5	71.1 ± 3.9	11.0 ± 10.8	6.4 ± 0.8	2.6 ± 0.1	0.11 ± 0.03	0.09 ± 0.01	0.05 ± 0.002
52.5	7.5	40	76.4 ± 5.3	69.1 ± 2.4	107.4 ± 2.5	88.2 ± 3.7	12.2 ± 0.8	1.0 ± 0.5	−2.1 ± 0.8	0.12 ± 0.02	0.13 ± 0.01	0.07 ± 0.02
32.5	7.5	60	74.3 ± 3.6	71.1 ± 6.6	107.7 ± 3.9	84.5 ± 2.1	10.9 ± 0.3	−0.1 ± 0.5	−5.6 ± 2.8	0.15 ± 0.01	0.15 ± 0.03	0.10 ± 0.01
85	15	0	80.7 ± 7.2	83.9 ± 2.8	98.2 ± 1.1	67.2 ± 0.5	23.9 ± 1.6	12.0 ± 0.6	9.8 ± 0.4	0.17 ± 0.05	0.14 ± 0.03	0.10 ± 0.01
65	15	20	68.5 ± 1.7	72.7 ± 8.5	88.1 ± 2.7	88.4 ± 1.3	20.1 ± 0.9	4.0 ± 6.3	2.0 ± 4.3	0.18 ± 0.01	0.15 ± 0.01	0.10 ± 0.01
45	15	40	67.0 ± 5.9	66.2 ± 0.8	90.9 ± 6.9	78.4 ± 3.0	16.2 ± 0.6	3.2 ± 0.4	−0.1 ± 0.3	0.19 ± 0.01	0.16 ± 0.02	0.11 ± 0.01
25	15	60	55.2 ± 11.4	64.0 ± 4.7	194.7 ± 14.6	0	15.1 ± 0.4	−0.6 ± 2.0	−5.6 ± 2.4	0.28 ± 0.04	0.23 ± 0.005	0.16 ± 0.02

(a) Size, PdI, and zeta potential (ZP) were measured by dynamic light scattering. Dynamic light scattering was performed in HEPES buffer. (b) Encapsulation efficiency was evaluated by Ribogreen® assay. The mean ± SD of three independent samples is shown. fLNPs; mRNA-free LNPs

Table S3. Particle properties of LNPs with different L/R ratios.

L/R ratio	Method	Size (nm) a)	Zeta potential (mV) a)	PdI a)	Encapsulation efficiency (%) b)
200	RtoU/Liq	88.3 ± 3.8	−2.0 ± 0.8	0.083 ± 0.002	95.3 ± 3.4
100	RtoU/Liq	105.7 ± 5.4	−1.5 ± 0.6	0.05 ± 0.03	93.7 ± 5.1
50	RtoU/Liq	133.4 ± 12.2	−5.1 ± 1.6	0.087 ± 0.002	49.5 ± 14.5
25	RtoU/Liq	213.4 ± 45.7	−27.2 ± 1.8	0.25 ± 0.02	0
50	EtD	57.6 ± 2.4	−1.8 ± 0.5	0.09 ± 0.01	86.7 ± 0.4
25	EtD	65.9 ± 6.6	−2.1 ± 0.8	0.08 ± 0.02	84.4 ± 0.8

(a) Size, PdI, and zeta potential (ZP) were measured by dynamic light scattering. Dynamic light scattering was performed in HEPES buffer. (b) Encapsulation efficiency was evaluated via Ribogreen® assay. The mean ± SD of three independent samples is shown.

Table S4. Particle properties of LNPs with different mixing conditions.

L/R ratio	Volume ratio (LNPs: mRNA)	Mixing method a)	Lipid conc. Before mixing (mM)	mRNA conc. Before mixing (mg/mL)	Lipid conc. After mixing (mM)	mRNA conc. After mixing (mg/mL)	Size (nm) b)	Zeta potential (mV) b)	PdI b)	Encap. efficiency (%) c)
100	1:9	Rapid	20	0.022	2	0.02	92.6 ± 4.4	−1.8 ± 0.3	0.10 ± 0.02	87.0 ± 1.0
100	5:5	Rapid	4	0.04	2	0.02	97.1 ± 2.7	−2.1 ± 0.2	0.09 ± 0.001	86.0 ± 1.5
100	9:1	Rapid	2.2	0.20	2	0.02	93.6 ± 3.3	−2.2 ± 0.2	0.10 ± 0.01	85.4 ± 0.7
100	1:9	Slow	20	0.022	2	0.02	90.4 ± 4.3	−2.2 ± 0.6	0.10 ± 0.01	85.1 ± 2.3
100	5:5	Slow	4	0.04	2	0.02	92.0 ± 4.9	−1.9 ± 0.6	0.10 ± 0.01	79.1 ± 3.8
100	9:1	Slow	2.2	0.20	2	0.02	89.4 ± 0.9	−2.4 ± 0.2	0.14 ± 0.02	23.1 ± 3.4
50	1:9	Rapid	20	0.044	2	0.04	124.4 ± 3.8	−4.7 ± 0.4	0.07 ± 0.02	72.7 ± 3.1
50	5:5	Rapid	4	0.08	2	0.04	121.3 ± 1.0	−4.3 ± 0.6	0.10 ± 0.01	52.3 ± 13.1
50	9:1	Rapid	2.2	0.40	2	0.04	120.4 ± 4.5	−5.2 ± 0.6	0.11 ± 0.01	51.8 ± 17.8
50	1:9	Slow	20	0.044	2	0.04	114.9 ± 3.5	−3.3 ± 0.2	0.09 ± 0.003	54.9 ± 2.8
50	5:5	Slow	4	0.08	2	0.04	120.5 ± 6.5	−4.4 ± 0.5	0.11 ± 0.01	39.1 ± 3.2
50	9:1	Slow	2.2	0.40	2	0.04	112.9 ± 5.4	−8.5 ± 1.1	0.14 ± 0.02	6.3 ± 5.6

(a) Rapid; vortex mixing and Slow; gentle pipetting. (b) Size, PdI, and zeta potential (ZP) were measured by dynamic light scattering. Dynamic light scattering was performed in HEPES buffer. (c) Encapsulation efficiency was evaluated via Ribogreen® assay. The mean ± SD of three independent samples is shown. The size distributions of these particles are shown in Figure S7

Table S5. Particle properties of LNPs prepared by various conditions.

Method a)	Buffer: EtOH	Flow rate	L/R ratio	Size of mRNA-free LNPs (nm) b)	PdI of mRNA-free LNPs b)	ZP of mRNA-free LNPs (mV) b)	Size of mRNA-LNPs (nm) b)	PdI of mRNA-LNPs b)	ZP of mRNA-LNPs (mV) b)	EE (%) c)
ME	1:1	15 mL/min	50	205.6 ± 5.1	0.13 ± 0.02	1.4 ± 0.2	233.4 ± 28.5	0.22 ± 0.01	−1.6 ± 0.2	0
ME	1:1	30 mL/min	50	195.9 ± 5.0	0.09 ± 0.03	1.3 ± 0.2	243.2 ± 5.6	0.15 ± 0.004	−0.9 ± 0.4	0
ME	1:1	45 mL/min	50	200.0 ± 5.5	0.05 ± 0.03	1.1 ± 0.04	215.6 ± 21.8	0.12 ± 0.02	−1.4 ± 0.4	0
ME	1:1	60 mL/min	50	185.2 ± 6.0	0.09 ± 0.01	1.2 ± 0.2	231.8 ± 10.1	0.14 ± 0.01	−0.7 ± 0.5	0
ME	3:1	15 mL/min	50	170.7 ± 12.4	0.16 ± 0.01	0.8 ± 0.3	171.2 ± 10.2	0.18 ± 0.01	−0.5 ± 0.4	7.4 ± 3.1
ME	3:1	30 mL/min	50	146.5 ± 9.2	0.13 ± 0.01	0.8 ± 0.1	154.5 ± 7.4	0.14 ± 0.01	−0.1 ± 0.3	34.3 ± 1.6
ME	3:1	45 mL/min	50	130.1 ± 8.1	0.10 ± 0.01	0.4 ± 0.3	153.6 ± 9.6	0.13 ± 0.02	−1.4 ± 0.4	0.6 ± 1.4
ME	3:1	60 mL/min	50	130.1 ± 8.6	0.11 ± 0.004	0.9 ± 0.3	150.2 ± 3.1	0.10 ± 0.01	0.1 ± 0.1	51.8 ± 1.8
ME	7:1	15 mL/min	50	158.9 ± 14.3	0.12 ± 0.01	1.1 ± 0.2	176.6 ± 4.5	0.09 ± 0.02	−0.6 ± 0.2	26.0 ± 3.4
ME	7:1	30 mL/min	50	132.1 ± 11.8	0.13 ± 0.003	1.0 ± 0.3	140.2 ± 14.1	0.13 ± 0.01	−0.3 ± 0.4	39.3 ± 2.6
ME	7:1	45 mL/min	50	124.4 ± 22.3	0.15 ± 0.02	0.5 ± 0.1	144.2 ± 0.8	0.16 ± 0.01	−0.5 ± 0.3	32.0 ± 1.4
ME	7:1	60 mL/min	50	125.7 ± 8.0	0.16 ± 0.02	0.3 ± 0.1	149.4 ± 6.9	0.14 ± 0.005	−0.7 ± 0.2	18.9 ± 2.6
MF	7:1	1 mL/min	50	92.2 ± 6.6	0.14 ± 0.01	0.7 ± 0.5	122.5 ± 7.2	0.09 ± 0.02	−0.5 ± 0.2	65.3 ± 1.0
MF	7:1	20 mL/min	50	63.9 ± 4.4	0.23 ± 0.01	0.6 ± 0.2	106.3 ± 8.8	0.11 ± 0.3	−0.6 ± 0.3	82.8 ± 1.0
BM	1:1	N.A.	100	215.7 ± 0.9	0.04 ± 0.01	1.1 ± 0.6	263.7 ± 9.2	0.11 ± 0.02	−1.0 ± 0.2	0
BM	3:1	N.A.	100	108.1 ± 0.9	0.06 ± 0.03	0.6 ± 0.1	130.8 ± 0.8	0.04 ± 0.01	0.1 ± 0.4	85.7 ± 1.0
BM	7:1	N.A.	100	126.8 ± 7.4	0.04 ± 0.01	0.0 ± 0.3	141.1 ± 2.3	0.05 ±	0.1 ± 0.3	85.0 ± 0.9

(a) ME; membrane emulsification, MF; microfluidic mixing, and BM; bulk mixing. (b) Measured by dynamic light scattering. (c) Encapsulation efficiency was evaluated via Ribogreen® assay. The mean ± SD of three independent samples is shown.

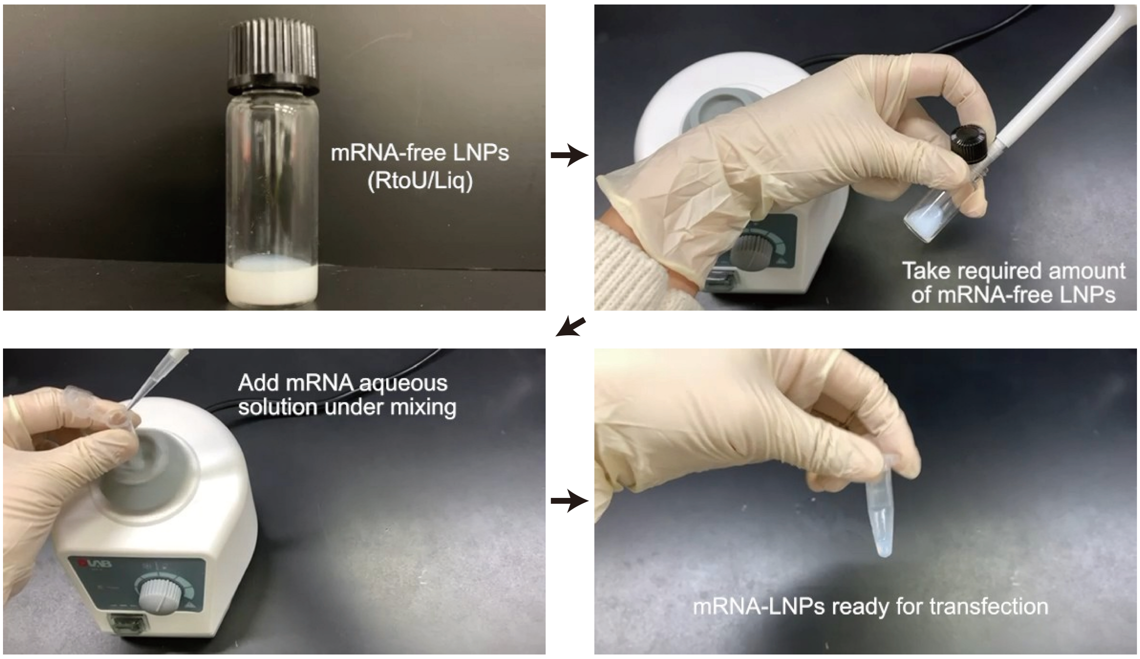

Caption of Movie S1 (separate file). A demonstration of how mRNA-LNPs are prepared using the mRNA-free LNPs(RtoU/Liq). The mRNA-free LNPs(RtoU) is a white translucent suspension. After transferring the required amount of mRNA-free LNPs into a plastic tube, the mRNA solution is added with mixing. This process yields mRNA-LNPs.

S5. References for Supporting Information

(1) Webster, E. R.; Peck, N. E.; Echeverri, J. D.; Gholizadeh, S.; Tang, W.-L.; Woo, R.; Sharma, A.; Liu, W.; Rae, C. S.; Sallets, A.; Adusumilli, G.; Gunasekaran, K.; Haabeth, O. A. W.; Leong, M.; Zuckermann, R. N.; Deutsch, S.; McKinlay, C. J. Discovery of a Peptoid-Based Nanoparticle Platform for Therapeutic MRNA Delivery via Diverse Library Clustering and Structural Parametrization. *ACS Nano* **2024**, *18* (33), 22181–22193.

(2) Tanaka, H.; Hagiwara, S.; Shirane, D.; Yamakawa, T.; Sato, Y.; Matsumoto, C.; Ishizaki, K.; Hishinuma, M.; Chida, K.; Sasaki, K.; Yonemochi, E.; Ueda, K.; Higashi, K.; Moribe, K.; Tadokoro, T.; Maenaka, K.; Taneichi, S.; Nakai, Y.; Tange, K.; Sakurai, Y.; Akita, H. Ready-to-Use-Type Lyophilized Lipid Nanoparticle Formulation for the Postencapsulation of Messenger RNA. *ACS Nano* **2023**, *17* (3), 2588–2601.

(3) Alabi, C. A.; Love, K. T.; Sahay, G.; Yin, H.; Luly, K. M.; Langer, R.; Anderson, D. G. Multiparametric Approach for the Evaluation of Lipid Nanoparticles for SiRNA Delivery. *Proc. Natl. Acad. Sci. U. S. A.* **2013**, *110* (32), 12881–12886.

(4) Simonsen, J. B. A Perspective on Bleb and Empty LNP Structures. *J. Control. Release* **2024**, *373*, 952–961.

(5) Neves, P.; Lopes, S. C. D. N.; Sousa, I.; Garcia, S.; Eaton, P.; Gameiro, P. Characterization of Membrane Protein Reconstitution in LUVs of Different Lipid Composition by Fluorescence Anisotropy. *J. Pharm. Biomed. Anal.* **2009**, *49* (2), 276–281.

(6) Nelson, S. C.; Neeley, S. K.; Melonakos, E. D.; Bell, J. D.; Busath, D. D. Fluorescence Anisotropy of Diphenylhexatriene and Its Cationic Trimethylamino Derivative in Liquid Dipalmitoylphosphatidylcholine Liposomes: Opposing Responses to Isoflurane. *BMC Biophys.* **2012**, *5* (1), 5.

(7) Tanaka, H.; Oasa, S.; Kinjo, M.; Tange, K.; Nakai, Y.; Harashima, H.; Akita, H. Temperature and PH Sensitivity of a Stabilized Self-Nanoemulsion Formed Using an Ionizable Lipid-like Material via an Oil-to-Surfactant Transition. *Colloids Surf. B Biointerfaces* **2017**, *151*, 95–101.

(8) Nakatani, A. I.; Mohler, C. E.; Hughes, S. Chain Conformation of Polymers Adsorbed to Clay Particles: Effects of Charge and Concentration. *Soft Matter* **2021**, *17* (28), 6848–6862.

(9) Feng, L.; Stuart, M. C.; Adachi, Y. Dynamics of Polyelectrolyte Adsorption and Colloidal Flocculation upon Mixing Studied Using Mono-Dispersed Polystyrene Latex Particles. *Adv. Colloid Interface Sci.* **2015**, *226* (Pt A), 101–114.

(10) Wang, L.; Shen, Y.; Yang, Y.; Lu, W.; Li, W.; Wei, F.; Zheng, G.; Zhou, Y.; Zheng, W.; Cao, Y. Stern-Layer Adsorption of Oligonucleotides on Lamellar Cationic Lipid Bilayer Investigated by Polarization-Resolved SFG-VS. *ACS Omega* **2017**, *2* (12), 9241–9249.

(11) Lai, E.; van Zanten, J. H. Real Time Monitoring of Lipoplex Molar Mass, Size and Density. *J. Control. Release* **2002**, *82* (1), 149–158.

(12) Gershon, H.; Ghirlando, R.; Guttman, S. B.; Minsky, A. Mode of Formation and Structural Features of DNA-Cationic Liposome Complexes Used for Transfection. *Biochemistry* **1993**, *32* (28), 7143–7151.
